# Ultrasound in Women's Health: Mechanisms, Applications, and Emerging Opportunities

**DOI:** 10.1002/adma.202520454

**Published:** 2026-02-05

**Authors:** Sarah B. Ornellas, Bilal Kizilaslan, Aastha Shah, Jason F. Hou, Yoonsoo Shin, Alejandra Hernandez Moyers, Claudia Lozano, Canan Dagdeviren

**Affiliations:** ^1^ Media Lab Massachusetts Institute of Technology Cambridge Massachusetts USA; ^2^ School of Medicine Acıbadem Mehmet Ali Aydınlar University İstanbul Türkiye; ^3^ Center for Biomedical Engineering School of Engineering Brown University Providence Rhode Island USA; ^4^ Harvard‐MIT MD‐PhD Program Harvard Medical School Boston Massachusetts USA

**Keywords:** point‐of‐care, portable technologies, ultrasound, women's health

## Abstract

Women's health remains inadequately served due to the historical predominance of males as the biological reference in medical research, leading to persistent sex‐based gaps in the understanding, diagnosis, and management of disease. As healthcare moves toward decentralization, e.g., through the collection of person‐generated health data, point‐of‐care diagnostics, and wearable devices, there is a critical need for tools tailored to women's unique conditions and presentations. Ultrasound technologies, recognized for their versatility and safety, have evolved from imaging to multifunctional platforms, with growing roles in diagnosis and therapy. Diagnostic ultrasound non‐invasively assesses anatomical features and functional information, and therapeutic ultrasound can perform targeted interventions, including neuromodulation, immunomodulation, thermal ablation, and drug delivery. By exploring the fundamental physical principles of ultrasound, including acoustic streaming, cavitation, and thermal interactions, and linking these mechanisms to cellular and tissue responses, this review highlights the capability of ultrasound to address female‐specific health disparities, especially in conditions that are undertreated or differentially expressed in women. Advancements in ultrasound technologies could significantly enhance clinical outcomes and improve the quality of life for women affected by conditions currently underserved by traditional medical interventions.

## Graphical abstract

As healthcare moves toward decentralization, ultrasound technologies are evolving from strictly imaging tools in clinical settings into versatile diagnostic and therapeutic platforms, with growing roles addressing women's health needs. This review highlights how ultrasound's underlying physical mechanisms can be harnessed to reduce disparities in women's health and improve outcomes for conditions historically underserved by conventional treatments.

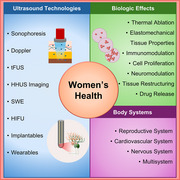

## 1 Introduction

Women account for half of the global population, yet only 1% of Research and Development funding for healthcare research is used for female‐specific conditions beyond oncology [1]. Historically, healthcare systems reflect an androcentric worldview, as women were often excluded from clinical trials until the NIH Revitalization Act mandated their inclusion [2] in 1993. The omission from medical research also influences how female bodies are treated when accessing care, as women's concerns are often dismissed, and their conditions are misdiagnosed [3, 4, 5]. The advent of Person‐Generated Health Data [6], decentralized trials that reduce barriers to clinical research [7], and Point‐of‐Care diagnostics [8] have the potential to address these challenges in women's health, strengthening the body of knowledge in female‐specific conditions and sex differentials in all diseases. Within the precision health ecosystem, “FemTech” refers to emerging digital technologies that help track lifestyle and hormone cycles, such as menstrual, pregnancy, and menopause trackers [9, 10], which collect multimodal biological data such as bodily fluids, basal body temperature, and physical activity [8, 10]. While FemTech applications have expanded the visibility of women's specific health needs, there is still a need for high‐resolution hardware capable of providing continuous, personalized physiological insights and/or disease management throughout a woman's life course. Future efforts in technology development aim to integrate screening, prevention and treatment of conditions across the lifespan, expanding the focus beyond reproductive health to include the management of leading causes of death or disability for women, such as cardiovascular diseases, cancers and neurological conditions [8, 11].

For those advancements to be realized, medical technologies that are safe, portable, and effective in diagnosis and treatment are crucial, and ultrasound technologies are well‐suited to this task. In this review paper, we highlight how ultrasound has been used across conditions that disproportionately or differentially affect women, and how opportunities emerge for it to become a leading technology in female‐focused medical research. On the diagnosis front, ultrasound stands out due to its potential to provide valuable and timely information when used appropriately by clinical practitioners, while also minimizing exposure to ionizing radiation [12]. This consideration is particularly critical in women of reproductive age, where minimizing radiation exposure supports both maternal and fetal health outcomes [11]. Therapeutic ultrasound, a relatively newer but rapidly expanding domain, offers versatile and minimally invasive treatment options [13]. While research and development are ongoing to bring those devices to the clinic with accurate and standardized dosimetry, therapeutic ultrasound has the potential to greatly improve access to care, leveraging portable and compact systems [14].

This review examines how ultrasound interfaces with the body and its potential to address sex differentials in healthcare. We begin by outlining the core mechanisms of action of ultrasound, exploring its diverse parameter space, and how it can be tailored for specific applications. We then cover studies in which ultrasound is applied to conditions that women are affected by more often or differentially, across the reproductive, cardiovascular, nervous, and multisystem domains. Across these sections, we highlight the advantages of ultrasound, including precise targeting, reduced systemic drug exposure, longitudinal monitoring, and improved access through point‐of‐care, wearable, and implantable formats. Finally, we discuss key translational challenges that will guide future work, including dosimetry and safety standards, manufacturability and workflow integration, and endpoints that capture sex‐specific responses.

## 2 Physical and Biological Interactions of Ultrasound with the Body

Ultrasound (US) is a versatile technique that can be utilized in various women's health applications across multiple diagnostic and therapeutic modalities. Ubiquitously used in prenatal care for fetal monitoring [15], ultrasound imaging can also be used to non‐invasively provide real‐time information about diverse body locations (e.g., breast [16], uterus [17], ovaries [18], brain [19], heart [20], thyroid [21]), from tissue and organ morphology [16] to blood flow [22] and functional monitoring [19]. Such information can be used for medical diagnosis or to guide minimally invasive therapies [23]. In recent years, therapeutic ultrasound has also gained attention, as it is capable of permanently and non‐invasively destroying diseased tissue (e.g., through thermal ablation [24] and histotripsy [25]) or temporarily affecting cellular mechanisms (e.g., neuro‐ and immunomodulation [26], sonoporation [27]). Importantly, ultrasound‐generating equipment is scalable and cost‐effective, enabling the tech to be translated from clinical to home use [28].

The ability of ultrasound to adapt across diagnostic and therapeutic contexts depends on how transducers are engineered to focus and deliver acoustic energy. For ultrasound imaging, short, low‐energy pulses do not modify the tissues, and information is extracted from the way the vibrations are reflected, scattered, or absorbed by the anatomical structures [29]. Transducer designs and coupling interfaces that match anatomic variation are critical for clinical effectiveness, and innovations such as soft and conformable transducers [30] have a high potential to address female‐specific needs. For therapeutic applications with increased energy deposition, focused and multi‐element arrays provide precise targeting of tissues, and the possibility to integrate patient‐specific sonoresponsive carriers aligns ultrasound with the goals of precision therapy [31]. Such approaches are especially relevant where targeted, localized therapy can reduce systemic exposure and adverse drug reactions, which are twice as likely to be experienced by women [32]. Across modalities, the miniaturization of transducers has facilitated new technologies that support longitudinal and preventive care, such as in wearable and implantable formats [33, 34, 35, 36].

### 2.1 Rationale for Ultrasound as a Versatile Platform for Women's Health

Ultrasound has long maintained a widespread relevance in medical applications as its interaction with the body is safe and radiation‐free. Since its advent in the 1940s, it has been used primarily in a diagnostic capacity for imaging cross‐sections of the brain and pelvic organs [37, 38]. Shortly thereafter, therapeutic applications of ultrasound began to be explored, especially High Intensity Focused Ultrasound (HIFU) [39, 40] and some initial investigations into sonophoresis [41]. The clinical adoption of therapeutic ultrasound, however, would lag for decades. Today, ultrasound is widely accepted as a reference modality for diagnostic purposes (imaging, Doppler, shear wave elastography), and therapeutic applications (precision therapy for ablation, including HIFU, histotripsy, neuromodulation, and targeted or transdermal drug delivery). With rapid advances in miniaturization of low‐power transducers and electronics, computational prowess, and advanced image reconstruction algorithms, these technologies offer unparalleled sophistication and control for probing human disease.

Beyond its broad diagnostic and therapeutic repertoire, ultrasound compares favorably with other imaging modalities in terms of accuracy, cost, and deployability at the point of care, which is particularly important in women's health. Across multiple indications, point‑of‑care ultrasound (POCUS) has demonstrated diagnostic accuracy comparable to benchmark modalities such as CT, MRI, PET, and angiography, while offering substantially lower cost and avoiding ionizing radiation [28, 42, 43, 44]. For example, ultrasound has shown similar sensitivity and specificity to CT or MRI for several abdominal and cardiovascular indications, yet at a fraction of the per‑patient cost and with shorter hospital stays [28, 42]. In emergency and pre‑hospital settings, out‑of‑hospital POCUS in ambulances and emergency departments has been associated with reduced time to necessary operations, lower overall treatment costs, and shorter admission lengths, by allowing rapid triage compared with waiting for CT or other resource‑intensive tests [28].

These advantages are particularly salient for women's health. Obstetric and gynecologic care in low‑ and middle‑income countries depends heavily on ultrasound for early detection of high‑risk pregnancies, evaluation of obstetric emergencies, and diagnosis of gynecologic pathology where CT or MRI are unavailable or unaffordable. Focused protocols such as FAST can be performed within minutes at the bedside to guide time‑critical decisions in trauma, while basic obstetric and cardiac ultrasound performed by midwives or mid‑level providers has shown good agreement with specialist sonographers and can meaningfully alter management in rural clinics [44]. At the same time, studies of pocket‑sized ultrasound devices show that, after brief training, adding handheld ultrasound to the physical examination improves diagnostic yield and reduces the need for further testing in both in‑ and outpatient settings, underscoring ultrasound's role as an extension of the clinician's senses rather than a replacement for high‑end imaging [43].

Ultrasound technology offers wide tunability for both sensing and actuation, making it an appealing choice for the design of systems for personalized medicine. Exploiting this functional parameter space (frequency, power, depth focusing) through better characterization of ultrasound physics in vivo will spawn new possibilities in the precise control and manipulation of ultrasound for closed‐loop health monitoring devices and non‐invasive therapeutic interventions. In this section, we provide a summary of the physical mechanisms of action of ultrasound in vivo. To support the discussion, we differentiate ultrasound‐related effects through parameters such as frequency (f), wavelength (λ), amplitude (A), and acoustic impedance (Z). A more in‐depth description of ultrasound parameters can be found in Note S1 and Table S1, where we present the relationship between physical phenomena that can be detected or created by ultrasound [45], and the resulting biological effects in vivo.

### 2.2 Physical Phenomena in Diagnostic Ultrasound

**FIGURE 1:**
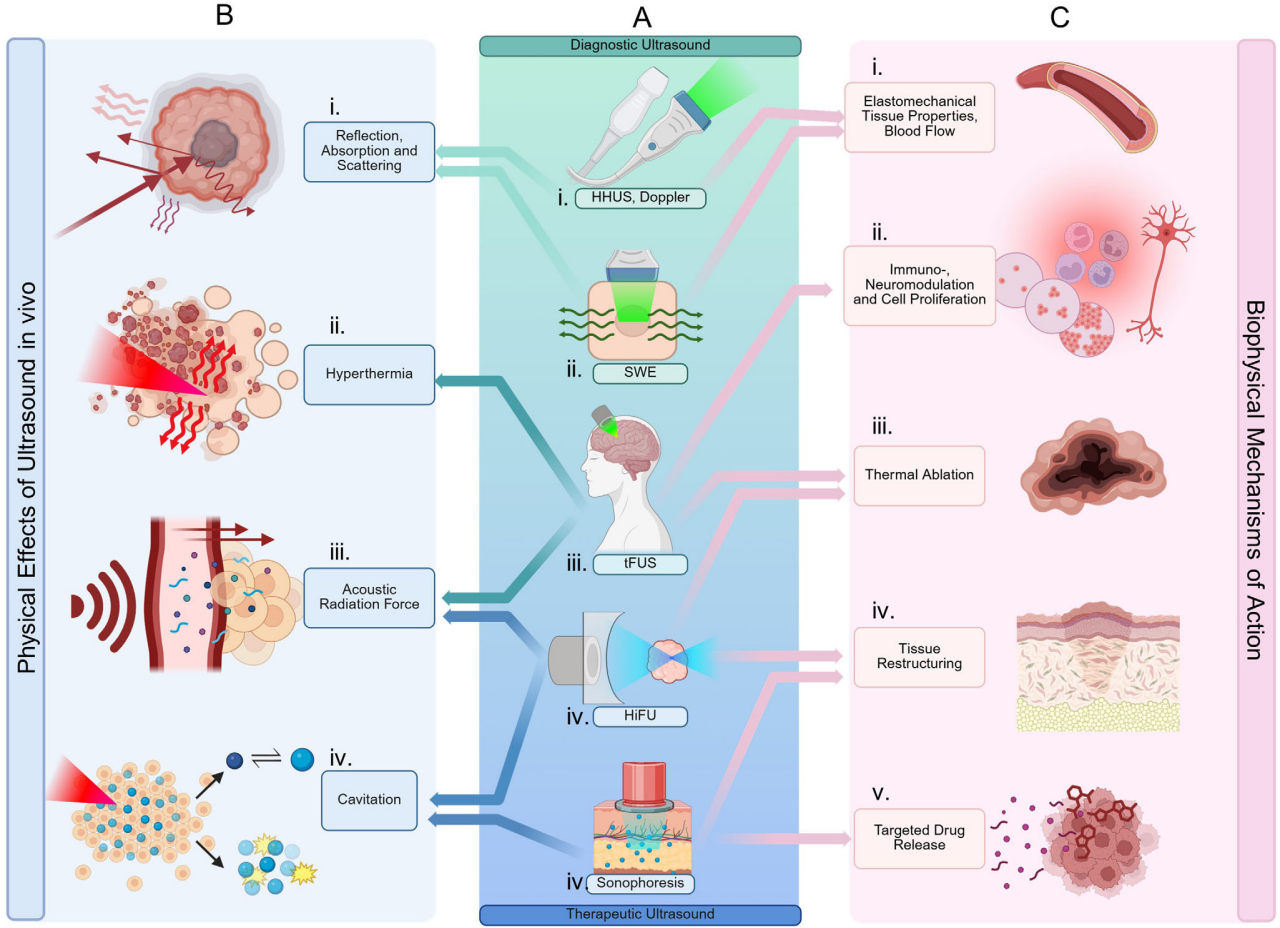
An infographic depicting the common modalities of medical and consumer ultrasound technologies (center, Panel A), as they relate to the underlying physical mechanism(s) (Panel B), and their bioeffect(s) in vivo (Panel C). A.i., Handheld ultrasound (HHUS) and A.ii., shear wave elastography (SWE) are based on the principles of differential reflection, absorption, and scattering of ultrasonic waves (B.i) by different types of tissue or blood flow. They are commonly used as diagnostic tools to assess tissue morphology through direct imaging, probe elasto mechanical tissue properties, and measure blood flow (C.i.). A.iii, Transcranial focused ultrasound (tFUS) has the capability to deliver a focused spot of ultrasound through the skull with varying intensities. Thermal (B.ii.) and mechanical (B.iii.) energy doses can be varied to achieve immuno‐ or neuromodulation (C.ii.), thermal ablation(C.iii.), or blood‐brain barrier opening for enhanced absorption of drugs (C.v.). A.iv., High‐intensity focused ultrasound causes extreme hyperthermia (B.ii.) and mechanical tissue damage through inertial cavitation (B.iv.). It is a commonly used modality for non‐invasive surgery (C.iii., C.iv.). A.v., Sonophoresis exploits acoustic cavitation effects (B.ii.) and localized heating (B.iv) to disrupt tissue structure. It is commonly used topically for skin restructuring (C.iv.) or for the transdermal absorption of drugs (C.v.). Figure created with BioRender.com.

Ultrasound waves are mechanical, longitudinal vibrations that propagate through a medium due to fluctuations in pressure and particle velocity. The behavior of these waves is governed by physical phenomena that relate frequency, wavelength, velocity, pulse duration, and pressure (or intensity). A change in any one of these quantities, brought about when a travelling ultrasonic pulse encounters a tissue boundary, forms the basis of ultrasound‐based sensing. The different interactions between ultrasound and boundaries (reflection, absorption, and scattering) form the foundation of diagnostic ultrasound imaging (Figure 1b).

Reflection occurs when an ultrasound wave encounters an interface with differing acoustic impedances. The magnitude of the reflected signal depends primarily on the impedance contrast, angle of incidence, and boundary width. B‐mode imaging, which stands for “Brightness mode”, is the standard imaging technique taken on Handheld Ultrasound Transducers (Figure 1a) and relies on the amplitude of the echoes for image generation. Large, smooth interfaces act as specular reflectors, whereas smaller or irregular boundaries generate partial or diffuse backscatter. Very small structures (<0.1λ) give rise to Rayleigh scattering, which produces weak but diagnostically useful echoes that contribute to image texture. In addition, reflections from moving scatterers show slight time or phase shifts, forming the basis of Doppler ultrasound (Figure 1a), which measures flow velocity and direction (Figure 1c) [22].

Absorption or attenuation of ultrasound may seem unintuitive as an aspect of imaging, as it presents a loss of energy returning to the transducer. However, it can be exploited in several ways. First, the measure of attenuation (dB/cm) of ultrasound by the tissue can provide important information about the tissue microstructure. It is commonly used for the detection of the fat composition of the liver or bone structures, a technique called Quantitative Ultrasound Imaging. Images created by this technique represent the ‘acoustic color’ of the tissue, a measurement typically complementary to standard B‐mode imaging. This is analogous to optical color in the sense that tissues scatter different ultrasound frequencies (low, mid, high) with different strengths and phase characteristics, just as different colors absorb/reflect different frequencies of light. For example, fat is fairly homogenous, and the backscatter power vs. frequency slopes off gradually. On the other hand, fibroglandular tissue attenuates mid‐range frequencies more strongly, thus presenting a sharper spectral slope.

Ultrasound backscatter imaging can also be exploited to reveal biological structures such as cells, fibers, or microbubbles. The reflection and attenuation patterns taken of the same insonified region from different probe angles and positions provide a signature of the intrinsic distribution in acoustic impedance inhomogeneities of the tissue. Analysis of such signals can be used to construct a speed of sound map of the tissue, which can be used to complement the standard B‐mode imaging to create a cleaner image or provide higher resolution images of the tissue microstructure.

Second, absorption of ultrasound at a focal point can cause local heating and tissue expansion, launching transverse shear waves through the tissue. These waves are approximately 1000 times slower than the primary longitudinal wave, and the rate of travel and attenuation of these shear waves (captured through a high frame rate on conventional imaging) can provide information on the elastic properties of the surrounding tissue, a technique called Shear Wave Elastography (SWE) (Figure 1b). SWE is primarily used in the evaluation of musculoskeletal tissue (Figure 1c).

### 2.3 Physical Phenomena in Therapeutic Ultrasound

While diagnostic ultrasound primarily focuses on imaging and tissue characterization, therapeutic ultrasound leverages the same physical principles to deliver energy in a controlled manner for biological modulation. By tuning frequency, power, duty cycle, and focal depth, ultrasound can induce a range of thermal and mechanical effects within targeted tissues. These effects underpin diverse therapeutic applications. The resulting bioeffects can be broadly divided into thermal and mechanical mechanisms. Thermal mechanisms include mild hyperthermia and high‐intensity ablation, both of which rely on the conversion of acoustic energy into localized heat. Mechanical effects, in contrast, arise from radiation forces, acoustic streaming, and cavitation, enabling precise manipulation of particles, cells, and tissue microenvironments. Together, these phenomena illustrate how ultrasound has evolved from a purely diagnostic tool into a versatile therapeutic platform capable of both tissue destruction and functional modulation. In the following sections, we summarize the principal therapeutic mechanisms of ultrasound (hyperthermia, acoustic radiation forces, and cavitation) and describe how these phenomena can be harnessed for diverse biomedical and clinical applications.

Ultrasound can be used to generate heat in a localized region through mechanical friction created by the microscopic oscillations of the viscous tissue as the wave concentrates within a given region (Figure 1a). Non‐linear effects, such as cavitation caused by large pressure amplitudes, can also contribute to local heating effects. Depending on exposure duration and intensity, the effects can yield either mild hyperthermia or irreversible thermal ablation. Mild hyperthermia (mHT), achieved by maintaining tissues at 40°C–45°C for extended periods, is used clinically to sensitize tumors to radiotherapy or chemotherapy. The temporary increase in temperature promotes vasodilation and oxygenation, enhancing the delivery and efficacy of oncolytic agents [46]. At moderate thermal doses, these effects remain reversible and can stimulate DNA repair pathways and systemic immune responses (Figure 1c). The tunability of ultrasound parameters (frequency, power, and focusing depth) enables selective heating of deep or superficial tissues [47, 48, 49]. Transcranial focused ultrasound (tFUS) (Figure 1b) is used to generate mild hyperthermia in addition to cavitation‐mediated disruption of the blood‐brain barrier, for the localized delivery of drugs to brain tumors. Details on quantification of the thermal dose through the CEM 43 parameter are provided in Note S1. At higher intensities and shorter exposure times, ultrasound energy can rapidly elevate local temperatures to induce irreversible thermal necrosis, a process known as thermal ablation (Figure 1c). This technique is an effective, non‐invasive method to create controlled thermal lesions for selective ablation of uterine fibroids [24, 50] and brain tumors [51], typically employing ultrasound frequencies in the range of 0.8–3.5 MHz. High frequencies (20 MHz) HIFU systems offer very limited tissue penetration and are used to treat superficial skin lesions with a high degree of precision.

Acoustic radiation forces refer to the net transfer of momentum from a travelling ultrasound wave to a particle or the medium (Figure 1a). It can be considered analogous to creating a DC impulse from an AC signal. The effect in vivo can occur due to absorption of the wave into the medium or due to a strong reflection. Depending on the acoustic parameters and target characteristics, radiation forces can produce distinct physical effects at both the tissue and cellular level. When a short (<1 ms), HIFU pulse is applied to a soft (∼50 kPa) viscoelastic tissue, it imparts a small deformation to the tissue surface. Tracking the displacement of this tissue using conventional B‐mode imaging provides rich information on the stiffness and viscoelastic properties of the tissue. This is the principle of acoustic radiation force impulse imaging (ARFI). For small particles, the effect of the ultrasonic ‘nudge’ serves to translate, aggregate, or trap particles. This effect is leveraged for trapping drugs for longer retention at a tumor, propelling drug‐loaded microbubbles across the blood‐brain barrier, or for a cell separation effect based on the mass and compressibility of the particle (acoustic tweezers) (Figure 1c). Acoustic streaming, or bulk movement of the medium (flow) has been observed through absorption of ultrasound within the medium (Eckart streaming) or through the creation of pressure gradients through standing waves. Streaming serves primarily as a mechanism to increase convective effects at the site of drug release, promoting increased uptake by the cell target.

Acoustic cavitation is defined as the nucleation, growth, oscillation, movement, and collapse of tiny bubbles when ultrasound is irradiated to a liquid (Figure 1a). The relationship between cavitation, frequency, and pressure can be understood by the Mechanical Index (MI), a unitless ultrasound metric that can predict the likelihood of cavitation to occur and is used by the FDA to assign Marketing Clearance of Ultrasound systems as diagnostic or therapeutic [52]. Further details can be found in Note S1. Cavitation can occur spontaneously when ultrasound at high intensities vaporizes dissolved gases in the tissue, or can be supported at lower ultrasound intensities through the intravenous administration of microbubbles or ultrasound contrast agents. In biomedical applications, cavitation manifests in three main forms that vary by acoustic intensity and frequency. Assisted cavitation occurs when ultrasound contrast agents, typically lipid‐coated microbubbles or gas vesicles, are introduced to lower the cavitation threshold and stabilize bubbles within the circulation. Their lipid shells prolong bubble lifetime up to 24 h, allowing targeted activation within tumor vasculature to enhance perfusion and the retention of systemically administered chemotherapies. Stable cavitation, by contrast, involves the nucleation and sustained oscillation of micron‐sized bubbles in phase with the ultrasound field, usually at frequencies above 1 MHz. These rhythmic pulsations generate localized microstreaming and shear stresses that can transiently increase cell membrane permeability or promote fluid mixing within tissues. At higher acoustic pressures and lower frequencies (0.05–1 MHz), inertial cavitation predominates: bubbles grow to unstable sizes during rarefaction cycles and collapse violently, releasing bursts of mechanical energy. This process can produce tissue disruption through histotripsy or, when modulated to milder levels, transiently enhance tissue permeability (Figure 1b), which is a mechanism exploited in sonophoresis for transdermal drug delivery [41] and in sonoporation for intracellular gene or nanoparticle transfer [27].

### 2.4 Materials for Acoustic Coupling and Device Integration

The efficacy of ultrasound diagnosis and therapy in women's health is fundamentally governed by the acoustic properties of the materials at the device‐tissue interface. Particularly for soft, curvilinear anatomy such as the breast and the abdomen, conformal and comfortable interfaces are critical to ensure the efficacy and adoption of the intervention. Soft biological tissues typically exhibit characteristic acoustic impedances ranging between 1.3 and 1.7 MRayl [29]; therefore, matching this impedance between the transducer and the target tissue is essential to minimize reflection losses. Materials‐based approaches encompass three main strategies: flexible or stretchable device components that enable proper contact of transducers on soft and curvilinear surfaces, interface materials that ensure robust acoustic coupling, and acoustic lensing strategies that customize wavefront shaping [30].

To address the need for flexible and stretchable device components, materials used for transducer packaging and integration have evolved to support hair‐thin or flexible form factors that conform to female anatomy (Table 1). For instance, the skin‐adaptive focused ultrasound (SAFU) device utilizes a flexible composite of piezoelectric lead zirconate titanate (PZT‐5H) and polydimethylsiloxane (PDMS). Its MEMS‐compatible fabrication process eliminates complex manual assembly, allowing for the consistent production of patches capable of hemodynamic monitoring [53]. For internal applications, the ImPULS system demonstrates the potential of flexible packaging materials to create hair‐thin, implantable piezoelectric stimulators that deliver therapeutic ultrasound to deep tissue targets with minimal invasive impact [35].

**TABLE 1:** Strategies for device‐soft tissue integration in Ultrasound devices.

Coupling Strategy	Material / Device	Composition	Primary Function & Advantage
1. Flexible or Stretchable Components	SAFU Device [53]	PZT‐5H and PDMS composite	Enables hemodynamic monitoring via a flexible patch that conforms to skin; MEMS fabrication ensures consistency.
	ImPULS System [35]	Flexible packaging materials	Creates hair‐thin, implantable stimulators for deep tissue therapy with minimal invasiveness.
2. Interface Materials (Coupling)	BAUS Hydrogel [54, 55]	Hydrogel‐elastomer hybrid	Mimics tissue impedance and water content (∼95%); prevents dehydration and maintains adhesion for ∼48 h.
	Shapeable Couplant [56]	Matrix with cotton fiber & TiO_2_	"Silly‐putty" like stress response allows fluid transition to fill irregular gaps, then re‐solidifies for robust coupling.
3. Wavefront Shaping & Integration	wf‐UMP [57]	Transducer array + MN patch	Integrates emission, coupling, and drug delivery (microneedles) into a single closed‐loop wearable system.
	Acoustic Holography [58]	Programmable Metamaterials	Uses acoustically transparent materials for dynamic beamforming and precise wave modulation.
	High‐Res Metamaterial [59]	Rotatable T‐shaped Structures	Enables ultrahigh phase resolution for precise steering and focusing via simple geometric rotation.

Regarding interface materials, distinct innovations have emerged to overcome the limitations of conventional liquid or gel‐based couplants, which often suffer from dehydration and poor structural integrity. Recent advances in soft matter engineering have led to robust, hydrogel‐based acoustic couplants that mimic the high water content (∼95%) and acoustic impedance of soft tissue. Wang et al. introduced a bioadhesive ultrasound (BAUS) device utilizing a tough hydrogel‐elastomer hybrid that maintains robust skin adhesion and low attenuation for up to 48 h [54, 55]. Beyond static hydrogels, Chen et al. developed an arbitrarily shapeable couplant—composed of a “silly‐putty” like matrix with cotton fiber and TiO_2_ fillers—that exhibits a stress‐triggered solid‐to‐fluid transition. This allows the material to flow into irregular gaps at the skin interface before re‐solidifying, ensuring seamless acoustic continuity [56].

Finally, advancements in wavefront shaping and orderly integration are realizing the vision of closed‐loop systems. The wearable focused ultrasound microneedle patch (wf‐UMP) exemplifies this by combining a stretchable conformal transducer, a bioadhesive hydrogel, and a dissolving microneedle (MN) patch for transdermal drug delivery [57]. Looking toward the future of precise wave manipulation, Zhang et al. have demonstrated reconfigurable dynamic acoustic holography using acoustically transparent and programmable metamaterials, opening new avenues for precise beamforming [58]. Combined with recent advances in metamaterials improving phase resolution by rotating subwavelength structures within a rectangular waveguide, precise beam steering and focusing can be integrated without bulky additions, further expanding the toolkit for targeted acoustic therapies [59].

### 2.5 Versatility of Ultrasound Systems

Taken together, diagnostic and therapeutic ultrasound systems demonstrate how acoustic energy can be precisely used to both visualize and modulate biological processes in a non‐invasive and radiation‐free way. Unlike optical or electromagnetic techniques, ultrasound provides deep tissue penetration and real‐time feedback, while its tunable parameters, such as frequency, power, and focusing depth, allow controlled transitions between sensing and actuation. Diagnostic modalities of ultrasound, including Doppler, elastography, and backscatter imaging, offer structural and functional information that can guide therapeutic interventions, whereas therapeutic ultrasound modalities enable local delivery of heat, mechanical stress, or therapeutic agents with sub‐millimeter precision. Ongoing advances in hardware miniaturization, wearable designs, and computational imaging are expanding the reach of ultrasound toward continuous monitoring and personalized medicine. As these technologies evolve, their integration with artificial intelligence, feedback‐controlled therapies, and multimodal imaging could transform ultrasound from a single‐purpose device into a dynamic interface for real‐time diagnosis and targeted treatment.

## 3 Ultrasound Use Cases in Women's Health Conditions across Body Systems

### 3.1 Women's Health Landscape

This section highlights case studies across diverse organ systems in which ultrasound technologies address conditions that significantly impact women's health and well‐being. Using a systems‐based approach, Tables 2, 3, 4 and 5 summarize sex‐specific prevalence and other important metrics for conditions in the nervous, reproductive, cardiovascular, and multisystem domains, using data from the Global Burden of Disease Study 2021 [60]. Subsequent subsections detail ultrasound's role in diagnosing and treating these conditions, with particular attention to innovations supporting targeted and tailored approaches in women's healthcare.

**TABLE 2:** Prevalence of sex‐influenced diseases among women and men in different age groups.

		Prevalance (per 100.000)							
System	Disease	15‐49		50‐74		75+		All Ages	
		Female	Male	Female	Male	Female	Male	Female	Male
Reproductive System	Gynecological Diseases (General)	62.166,12	NA	27.976,40	NA	18.734,44	NA	38.906,31	NA
	Breast Cancer	246,55	2,68	1.420,82	27,14	2.123,16	41,03	516,87	8,09
	Ovarian Cancer	26,80	NA	73,05	NA	50,80	NA	31,09	NA
	Uterine Fibroids	730,46	NA	3.881,71	NA	4.370,81	NA	3.040,34	NA
	Endometriosis	1.079,91	NA	145,99	NA	0,00	NA	566,51	NA
Cardiovascular System	Cardiovascular Disease (General)	2.847,01	2.786,42	18.715,72	22.146,04	50.762,12	56.428,67	7.733,33	7.778,67
	Hypertension (Hypertensive Heart Disease)	27,94	29,15	353,81	372,01	1.958,44	1.768,97	173,03	144,01
Nervous System	Alzheimer's Disease	17,40	14,82	1.365,98	1.045,64	14.486,22	9.986,60	918,20	524,15
	Multiple Sclreosis	31,13	15,12	65,76	34,38	64,07	30,81	32,32	15,58
	Parkinson's Disease	9,89	14,56	307,07	438,93	1.522,72	2.176,36	135,52	162,62
	Migraine	25.345,02	15.087,45	18.726,40	10.794,30	8.754,92	5.476,37	18.444,79	10.940,84
	Depression	6.220,98	4.035,19	7.534,55	5.119,61	6.493,94	4.835,62	5.118,72	3.312,23
Multisystem	Endocrine, metabolic, blood, and immune disorders	8.739,82	3.941,90	13.962,59	7.144,31	12.613,25	6.319,14	8.249,11	3.824,57
	Diabetes	4.055,51	4.695,23	16.362,76	18.146,42	22.221,17	25.497,91	6.480,55	6.840,48
	Rheumatoid Arthritis	228,34	81,02	807,83	332,90	1.000,14	527,64	329,99	125,01

Several diseases in Tables 2, 3 are intrinsically female‐specific (e.g., ovarian cancer, uterine fibroids) or more prevalent in women (e.g., depression rheumatoid arthritis) [60, 61, 62]. For other conditions, including diabetes and hypertension, risks are amplified across the lifetime due to hormonal fluctuation and reproductive events, such as pregnancy and menopause [63, 64]. Additionally, many conditions present with sex‐specific differences in symptom manifestation or progression [4, 65, 66], influencing mortality rates (Table 4). Recognizing these distinctions is crucial for understanding how ultrasound can be leveraged to support both generalized, efficient diagnostic and therapeutic care and targeted approaches that are sex‐specific.

**TABLE 3:** Incidence of sex‐influenced diseases among women and men in different age groups.

		Incidence (per 100.00)							
System	Disease	15‐49		50‐74		75+		All Ages	
		Female	Male	Female	Male	Female	Male	Female	Male
Reproductive System	Gynecological Diseases (General)	31.348,61	NA	13.188,26	NA	8.056,56	NA	19.416,26	NA
	Breast Cancer	28,81	0,32	141,92	3,30	194,89	4,91	52,97	0,98
	Ovarian Cancer	4,40	NA	19,72	NA	28,12	NA	7,60	NA
	Uterine Fibroids	504,41	NA	22,77	NA	3,59	NA	256,88	NA
	Endometriosis	176,49	NA	0,91	NA	0,00	NA	87,67	NA
Cardiovascular System	Cardiovascular Disease (General)	230,51	283,95	1.934,88	2.477,84	6.293,07	6.702,84	820,28	872,78
	Hypertension (Hypertensive Heart Disease)	NA	NA	NA	NA	NA	NA	NA	NA
Nervous System	Alzheimer's Disease	5,30	4,48	238,60	175,20	2.435,59	1.779,28	157,47	92,07
	Multiple Sclreosis	1,74	0,95	0,42	0,45	0,31	0,34	1,00	0,60
	Parkinson's Disease	1,67	2,42	36,13	52,30	141,01	243,80	14,57	19,25
	Migraine	1.710,48	1.028,79	644,03	408,70	275,70	204,07	1.409,72	877,75
	Depression	6.837,27	4.314,06	7.695,86	4.947,88	7.308,42	5.176,29	5.584,95	3.481,36
Multisystem	Endocrine, metabolic, blood, and immune disorders	1.370,77	639,53	2.310,40	1.159,44	2.354,66	1.147,54	1.375,00	641,75
	Diabetes	278,33	324,58	702,47	731,31	178,19	197,04	298,77	320,63
	Rheumatoid Arthritis	16,76	6,06	36,75	18,65	25,69	21,81	17,69	7,70

**TABLE 4:** Mortality of sex‐influenced diseases among women and men in different age groups.

		Mortality (per 100.00)							
System	Disease	15‐49		50‐74		75+		All Ages	
		Female	Male	Female	Male	Female	Male	Female	Male
Reproductive System	Gynecological Diseases (General)	0,19	NA	0,36	NA	1,27	NA	0,23	NA
	Breast Cancer	6,64	0,08	42,50	1,03	103,30	2,75	16,80	0,33
	Ovarian Cancer	1,29	NA	13,22	NA	29,20	NA	4,72	NA
	Uterine Fibroids	0,05	NA	0,08	NA	0,22	NA	0,05	NA
	Endometriosis	0,00	NA	0,00	NA	NA	NA	0,00	NA
Cardiovascular System	Cardiovascular Disease (General)	21,50	41,65	366,61	590,58	3.372,22	3.840,69	233,41	258,55
	Hypertension (Hypertensive Heart Disease)	1,31	1,56	28,77	28,80	302,39	244,39	19,69	14,08
Nervous System	Alzheimer's Disease	0,05	0,04	20,10	14,91	689,90	418,55	33,71	15,83
	Multiple Sclreosis	0,10	0,05	0,72	0,46	1,30	0,86	0,26	0,14
	Parkinson's Disease	0,03	0,07	4,35	6,65	78,10	136,49	4,27	5,55
	Migraine	NA	NA	NA	NA	NA	NA	NA	NA
	Depression	NA	NA	NA	NA	NA	NA	NA	NA
Multisystem	Endocrine, metabolic, blood, and immune disorders	0,67	0,74	3,72	4,28	24,12	23,70	2,31	2,15
	Diabetes	2,70	3,40	49,80	54,17	230,66	241,35	21,87	20,11
	Rheumatoid Arthritis	0,05	0,02	1,26	0,65	8,10	5,38	0,64	0,31

**TABLE 5:** Disability‐Adjusted Life Years (DALYs) of sex‐influenced diseases among women and men in different age groups. Data from the Global Burden of Disease Study 2021 [60].

		DALYs (per 100.00)							
System	Disease	15‐49		50‐74		75+		All Ages	
		Female	Male	Female	Male	Female	Male	Female	Male
Reproductive System	Gynecological Diseases (General)	1.143,57	NA	538,24	NA	284,05	NA	705,44	NA
	Breast Cancer	341,71	4,18	1.337,38	31,25	1.394,47	38,92	515,13	9,62
	Ovarian Cancer	66,44	NA	383,43	NA	382,14	NA	131,31	NA
	Uterine Fibroids	5,08	NA	4,54	NA	2,88	NA	3,63	NA
	Endometriosis	52,12	NA	13,10	NA	0,00	NA	52,12	NA
Cardiovascular System	Cardiovascular Disease (General)	1.257,83	2.228,48	10.417,06	16.916,50	41.683,96	50.414,33	4.691,75	6.158,77
	Hypertension (Hypertensive Heart Disease)	66,97	78,44	771,66	794,14	3.595,11	771,66	351,57	293,95
Nervous System	Alzheimer's Disease	5,35	4,45	757,75	556,52	10.343,16	6.593,49	605,51	316,31
	Multiple Sclreosis	13,81	7,02	36,67	21,92	31,84	19,09	16,10	8,59
	Parkinson's Disease	3,39	5,70	146,17	220,34	1136,15	1.977,99	81,37	107,89
	Migraine	936,45	572,20	713,23	428,75	311,36	202,98	683,73	416,60
	Depression	1.079,73	703,65	1.219,88	817,28	1.013,67	746,68	867,75	560,97
Multisystem	Endocrine, metabolic, blood, and immune disorders	177,96	97,07	327,08	230,41	472,01	393,95	198,69	127,55
	Diabetes	401,21	472,77	2.714,89	2.998,36	4.758,20	5.287,45	987,75	1.012,78
	Rheumatoid Arthritis	33,39	12,55	136,66	60,25	219,04	132,09	55,37	22,69

Disability‐adjusted life years (DALYs), which are covered in Table 5, are a unifying metric that captures both premature mortality and years lived with disability. For the 20 major causes of disease burden captured in the Global Burden of Disease Study 2021, DALYs were higher for women in morbidity‐driven conditions, such as mental, musculoskeletal, and neurological disorders, while males are disproportionately affected in mortality‐heavy conditions, such as COVID‐19, road injuries, stroke, and liver diseases [67]. Differences between females and males often emerge in adolescence and intensify over time, as women experience more chronic disability and men, early mortality [67]. Such observations, paired with ageing populations across the world, highlight the need for women's health research to not only focus on reproductive conditions, but also on those that affect women across their lifetime. In this context, ultrasound emerges as a versatile tool, capable of supporting early detection, monitoring of chronic conditions, and treatment strategies tailored to sex‐specific trajectories.

### 3.2 Conditions of the Reproductive System

**FIGURE 2:**
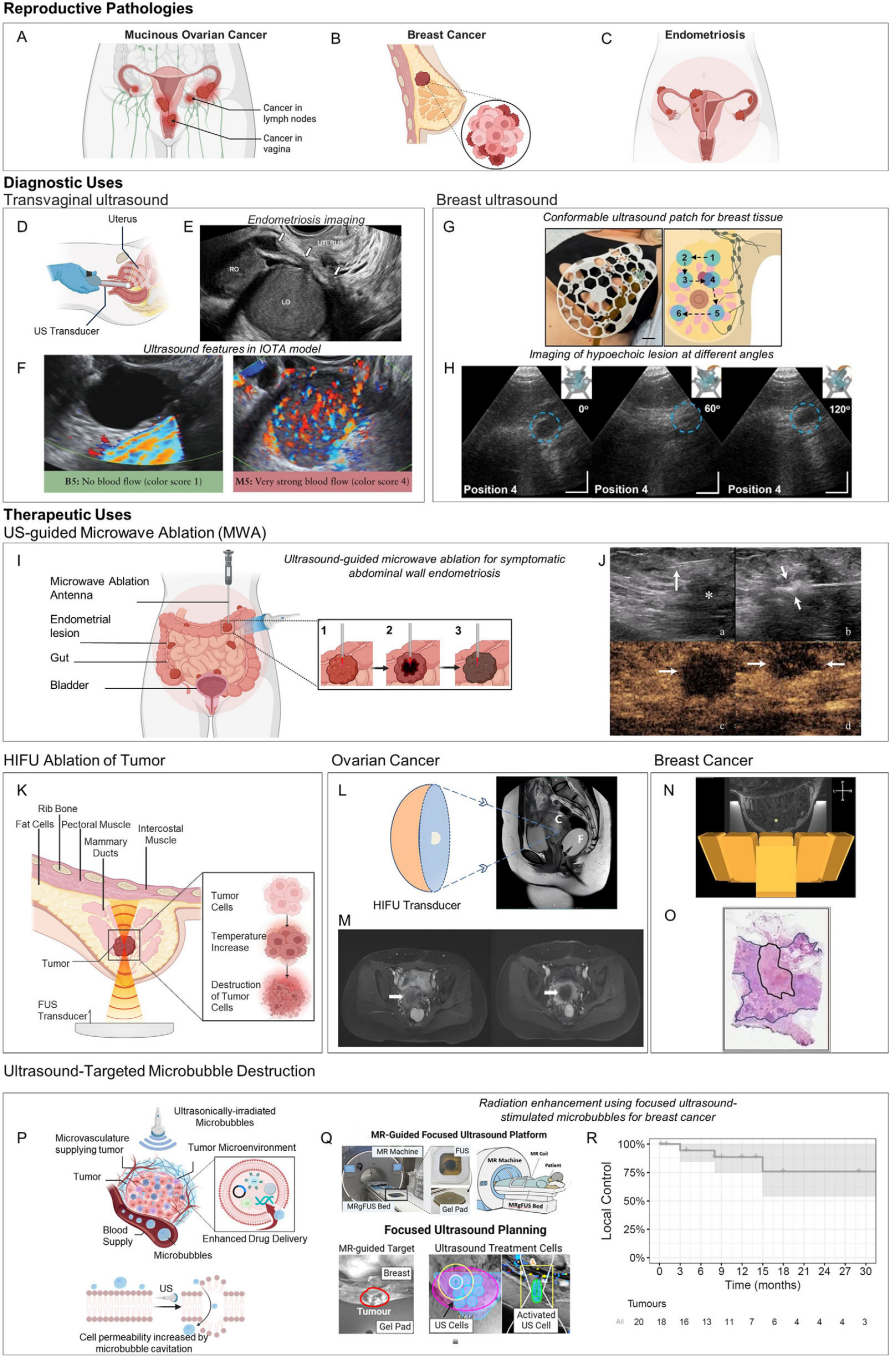
(A) Schematic of mucinous ovarian cancer, an epithelial subtype often diagnosed at an advanced stage with regional spread, contributing disproportionately to morbidity and mortality in women. (B) Schematic of breast cancer, the most frequently diagnosed malignancy in women. (C) Schematic of endometriosis, a female‐prevalent gynecologic disorder associated with pelvic pain, infertility, and diagnostic delay. (D) Schematic of transvaginal ultrasound (TVUS), a high‐resolution pelvic imaging approach used to evaluate the uterus, endometrium, ovaries, adnexal masses, and early pregnancy. (E) TVUS identifying retrouterine endometriosis (white arrows), with lesions adhered to the uterus and ovaries. LO, left ovary; RO, right ovary. (F) Ultrasound images illustrating example features classified by the International Ovarian Tumor Analysis (IOTA) rules. B5: No Blood Flow, benign feature. M5: Very strong blood flow, malignant feature. (G) Picture of wearable ultrasound breast patch on a female subject, which contains a miniature ultrasound element that can be rotated along a honeycomb patch, scale bar 2 cm (left), and schematic of the scanning sequence at different positions within the patch (right). (H) Ultrasound images taken by the element at position 4, when rotated at different angles (0°, 60°, and 120°). The blue dashed circle identifies a hypoechoic lesion. Scale bars 1 cm. (I) Schematic of ultrasound‐guided microwave ablation (US‐MWA) for deep endometriotic lesion, illustrating lesion targeting and the ablation sequence (1‐placement, 2‐thermal coagulation, 3‐postablation change). (J) Contrast‐enhanced Ultrasound (CEUS) is used to evaluate MWA efficacy. (a) A needle (white arrow) injects isolation fluid on the lesion vicinity (asterisk). (b) A hyperechoic cloud is formed on the lesion vicinity indicating successful MWA. (c‐d). CEUS after treatment shows no enhancement in the treated area, indicating complete ablation. (K) Schematic of high‐intensity focused ultrasound (HIFU) ablation of a breast tumor, illustrating focal energy deposition and heat‐induced destruction of tumor cells as a noninvasive local therapy. (L) Schematic of patient positioning for high‐intensity focused ultrasound (HIFU) ablation of ovarian mucinous carcinoma. C marks the target cancer lesion; F indicates the rectal Foley‐catheter balloon filled with degassed water used for acoustic coupling. (M) Contrast‐enhanced pelvic magnetic resonance imaging (MRI) before (left) and after (right) HIFU for recurrent ovarian cancer at the vaginal stump. The pretreatment scan shows an enhancing mass consistent with recurrence, whereas the post‐HIFU scan shows the absence of enhancement within the lesion, indicating successful ablation. White arrows indicate the tumor lesion. (N) MR image (T1‐weighted) of a healthy volunteer, with a schematic display of the ultrasound elements around the breast (transversal view). The green dot represents a sonication focus. P: Posterior; A: Anterior, L: Left; R: Right. (O) Microscopic picture of a hematoxylin and eosin staining after MR‐guided focused ultrasound of a breast tumor, showing treatment‐related necrosis. The tumor area is delineated by the blue line, and the tumor necrosis area is delineated by a black line. (P) Schematic of ultrasound‐targeted microbubble destruction (UTMD) for enhanced drug delivery, where sonicated microbubbles in the tumor microvasculature cavitate and increase vascular and cell‐membrane permeability. (Q) MR‐guided focused ultrasound workflow with microbubble therapy, showing the integrated MRI–FUS setup (patient in prone position, target coupled via a gel pad) and the treatment planning. After the tumor region is identified by MR imaging (red circle), ultrasound cells are placed over the entire target region. (R) Kaplan–Meier curve for local control of target breast tumors after radiotherapy enhanced with ultrasound‐stimulated microbubbles. The solid line shows the estimated local‐control probability, the shaded band is the 95% confidence interval, downward steps mark local failures, short vertical ticks indicate censored cases, and numbers at risk are listed beneath the x‐axis. Panels A, B, C, D, I, K, P were created with BioRender.com. Panel E adapted with permission [78] 2021, Oxford University Press. Panel F adapted with permission [73] 2013, John Wiley and Sons. Panels G and H adapted with permission [36] 2023, The American Association for the Advancement of Science. Panel J adapted with permission [23] 2022, Sage Publications. Panels L and M adapted under CC BY License [77] 2024, Frontiers Media SA. Panel N adapted with permission [79] 2013, Springer Nature. Panel O adapted with permission [80]. 2016, Springer Nature. Panels Q and R adapted under CC BY License [81] 2024, PLOS.

Conditions of the female reproductive system can reduce quality of life, through substantial disability from chronic gynecologic disease, and contribute to high mortality, especially through cancers that primarily affect women [68]. Intrinsic sex differences are shaped by cyclic estrogen–progesterone signaling, a tissue‐specific immune–inflammatory milieu, and life transitions such as pregnancy and menopause, which together influence susceptibility, symptom expression, and treatment response [66]. Ovarian cancer (Figure 2a) leads to a disproportionate amount of deaths due to its late presentation, while breast cancers remain the most frequently diagnosed malignancy in women worldwide (Figure 2b) [69]. In parallel, benign yet symptom‐intense conditions, including endometriosis (Figure 2c) and endometrial (uterine) polyps, cause pain, abnormal bleeding, infertility, and multi‐year diagnostic delays that erode quality of life [70, 71]. These patterns lead to delayed diagnosis, underscoring the need for sex‐specific, quantitative biomarkers of pelvic physiology (e.g., lesion vascularity, tissue stiffness, blood‐flow patterns) and for targeted interventions.

#### 3.2.1 Ovarian Cancer Diagnosis and Treatment

Ovarian cancer (OC) is the seventh most common malignancy in women and the eighth leading cause of cancer‐related death among women worldwide [72]. Ultrasound is frequently the first‐line imaging modality when ovarian pathology is suspected. It allows for detailed assessment of ovarian morphology, including size and internal architecture, which helps clinicians determine whether a mass is likely benign or potentially malignant (American Cancer Society). The use of models recommended by the International Ovarian Tumor Analysis (IOTA) [73] have allowed for the retention of high diagnostic accuracy of transvaginal ultrasound (TVS) (Figure 2d–f). These models have demonstrated high sensitivity and specificity in detecting both borderline and malignant ovarian tumors regardless of examiners experience [74]. Given the affordability and ubiquity of ultrasound, their prompt and accurate use can help make detection and risk stratification of ovarian tumors more accessible and cost‐effective across diverse healthcare settings

Standard treatment for ovarian cancer includes resection surgery, chemotherapy, and other combination therapies. However, recurrence remains a significant challenge, occurring in approximately 25% of patients with early‐stage disease and in over 80% of those with advanced‐stage disease [75]. This often requires further invasive surgeries to try to remove metastatic tumors. Moreover, surgical options may be limited for certain patients due to hard‐to‐reach tumors or other clinical factors.

High‐intensity focused ultrasound (HIFU), a noninvasive thermal ablation technique (Figure 2l), presents a promising adjunct or alternative treatment for difficult‐to‐treat tumors. Feasibility and clinical studies have shown that HIFU, when combined with chemotherapy, can reduce tumor burden and offer a viable approach for treating recurrent or surgically challenging ovarian cancer [76, 77]. In a case study, HIFU enabled successful targeted thermal ablation of the vaginal tumor tissue without causing damage to adjacent structures and minimizing off‐target effects [77] (Figure 2m).

#### 3.2.2 Breast Cancer Diagnosis and Treatment

In addition to ovarian cancer, ultrasound has been used in the diagnosis and management of breast cancer. Breast cancer is the most commonly diagnosed malignancy among women worldwide, but thanks to earlier diagnosis and better treatment, its mortality has continuously decreased [72]. Ultrasound is widely utilized as a first‐line and adjunct imaging technique to evaluate abnormalities found during physical evaluation, complement mammography, and guide biopsies. Ultrasound is a powerful tool in diagnosis, and it has been shown that in conjunction with mammography, it is equivalent to MR imaging in evaluating the extent of invasiveness of certain carcinomas [16]. For lobular carcinoma (ILC) and invasive ductal carcinoma (IDC), US showed even greater sensitivity than mammography alone [16]. In addition, compared with other screenings, annual ultrasound screening is the most cost‐effective screening in high‐risk populations, as assessed by a risk‐based breast Cancer Screening Program in the Urban Hebei Province [82].

Current work in ultrasound focuses on creating wearable and conformable interfaces, which could be used outside of hospital settings and allow for more frequent and accessible testing [83]. A novel wearable ultrasound breast patch was able to image cysts around 0.3 cm in vivo, which demonstrates its feasibility as a user‐friendly, at‐home breast cancer screening system (Figure 2g,h) [36]. Future implementation of image acquisition standardization, big data and AI integration into conformable ultrasound electronics could help enable such devices to identify edge cases that might be missed with current US acquisition methods [30].

Similar to ovarian cancer treatment, HIFU has been investigated as a noninvasive ablation technique for breast cancer, allowing for precise thermal ablation of tumor tissue without the need for surgery (Figure 2k). A clinical study using a dedicated MR‐HIFU breast platform (Figure 2n) [79] demonstrated by histopathology tumor necrosis of targeted locations (Figure 2o), with a significant correlation of the applied energy and the size of the necrosis, and a maximum temperature achieved around 61°C [80]. The treatment was deemed safe and feasible, with no adverse effects of skin burns or redness observed. Current phase I clinical trials [84] are evaluating the efficacy and safety of an MRgFUS. They are using the Muse system, an MRgFUS device developed by the University of Utah that uses a custom breast MR coil and an ultrasound transducer capable of targeting nearly any ablation zone in the breast, along with a customized comfort table that allows patients to lie face‐down throughout the procedure. These advances highlight the promise of HIFU as both a stand‐alone treatment and a complementary therapy, particularly for patients who are resistant to conventional treatments, want to retain mammary tissue, or prefer not to undergo extensive surgery.

In addition to ablation, ultrasound has also shown promise in enhancing therapeutic efficacy when combined with adjuvant modalities. As covered in the Physical Phenomena section, cell permeability can be increased through ultrasound irradiation of microbubbles, which then cavitate and provide openings through which drugs can more easily permeate the surrounding tissue, e.g., the tumor microenvironment (Figure 2p). A Phase 1 clinical trial demonstrated that ultrasound‐stimulated microbubbles can increase tumor sensitivity to radiotherapy in breast cancer patients, potentially improving treatment [81] (Figure 2q). The proposed mechanism of action revolves around pro‐apoptotic signaling and vascular disruption triggering when a microbubble‐sensitized tumor is treated with radiation, enhancing treatment effectiveness [85]. During the clinical trial, they observed that 83% of tumors achieved partial or complete responses, and the local control rate at 2 years was 76% [81] (Figure 2r). These developments solidify ultrasound not only as a cost‐effective diagnostic but also as an evolving therapeutic modality, with the potential to increase options of less invasive treatment and improve outcomes in breast cancer management.

#### 3.2.3 Management of Endometriosis

Besides cancer, there are “benign” diseases that impair quality of life with hard‐to‐treat symptoms. Endometriosis is a chronic gynecological condition where endometrial tissue is found outside the uterus, leading to pelvic pain, infertility, and many other symptoms. Historically, diagnosis required laparoscopic visualization with histopathology, which causes significant delays in diagnosis. More recently, transvaginal ultrasound (Figure 2d) has emerged as a non‐invasive diagnostic tool with the potential to replace diagnostic laparoscopy (Figure 2e) [78], with reported accuracies of ∼91% for ovarian endometriosis and ∼86% for deep infiltrating endometriosis (DIE) [86].

To treat abdominal wall endometriosis (AWE), while surgical excision remains the conventional treatment, minimally invasive treatments are currently being explored to improve accessibility and recovery times of treatment. HIFU has demonstrated promising results in a cohort of 51 women with AWE, achieving low recurrence rates (3.9% at 48 months) and minimal complications [87]. However, a more accessible treatment option is microwave ablation (MWA) (Figure 2i), a technique used in other gynecological issues such as uterine fibroids. Ultrasound can be used in conjunction with MWA for the localization of lesions pre‐treatment and monitoring of tissue post‐treatment. In their first experience, Li et al. [23] used ultrasound, MRI, and US‐guided biopsy to confirm the diagnosis of AWE and then contrast‐enhanced ultrasound (CEUS) to instantly evaluate treatment and during follow‐up visits. CEUS was used to confirm complete ablation (absence of perfusion) immediately post‐treatment (Figure 2j). Patients experienced significant pain reduction: symptoms pre‐operation ranged 4–6 in the VAS scale and decreased to 0–2 post‐treatment and lesion volume reductions (ranged: 16.6%–100%). US‐guided MWA enables real‐time visualization, minimally invasive treatment, and effective reduction of symptoms of AWE. Compared to conventional surgery and HIFU, US‐guided MWA provides a minimally invasive, easily available technique for the treatment of AWE.

#### 3.2.4 Hormonal Transitions of the Female Body throughout Life

In terms of physiology, the female body is fundamentally distinct from the male body due to dynamic, sex‐specific hormonal fluctuations that govern tissue mechanics, vascular physiology, and systemic metabolism throughout the lifespan. Unlike the relatively static male biological baseline, the female body undergoes cyclic remodeling mainly driven by estrogen and progesterone, which significantly influence ultrasound‐tissue interactions. Hormonal surges of estrogen and progesterone modulate collagen cross‐linking, water content, and vascular compliance, directly altering the acoustic impedance and viscoelasticity of soft tissue [88]. The biological differences also contribute to measurable variation between women and men in attenuation patterns, tissue perfusion, and cavitation thresholds, all of which shape diagnostic and therapeutic ultrasound performance (detailed physical explanations are provided in Section 2.)

This interplay is most pronounced during pregnancy, a state of extreme hemodynamic and anatomical adaptation. During pregnancy, endocrine shifts and structural changes such as increased uterine volume, cervical remodeling, placental vascular development, and alterations in myometrial conductivity directly influence acoustic propagation and tissue viscoelasticity. This physiological complexity creates a unique window for technological intervention. Recent systematic work has highlighted the expanding role of tele‐ultrasound, handheld, and even self‐operated ultrasound systems in pregnancy care, demonstrating that remote and decentralized scanning can be both feasible and clinically informative across diverse settings, including rural and low‐resource environments [89]. Moreover, emerging AI‐assisted ultrasound platforms are further enhancing maternal–fetal assessment by automating fetal biometry, anomaly detection, and image standardization, thereby reducing operator dependence and improving diagnostic reliability even in low‐resource settings [90]. Evaluation of placental structure, placental perfusion, and cervical biomechanics with ultrasound is still central to predicting preterm birth and identifying placental abnormalities, with ongoing development of quantitative metrics to support early risk stratification.

Conversely, the cessation of this hormonal flux during menopause presents a different set of challenges, particularly for skeletal integrity, marking a critical hormonal transition. As estrogen levels drop, the resulting acceleration in bone turnover leaves millions of women disproportionately vulnerable to fragility fractures compared to their male counterparts [91]. While Dual‐energy X‐ray Absorptiometry (DXA) remains the diagnostic gold standard, its reliance on ionizing radiation and hospital‐based infrastructure makes it unsuitable for the kind of frequent, preventative monitoring required to track rapid physiological changes [92]. This limitation highlights the unique value of Quantitative Ultrasound (QUS): unlike DXA, QUS offers a radiation‐free window into bone quality that can be accessed outside the clinic; however, QUS is not enough for continuous monitoring [92]. To make this continuous monitoring a reality, the technology must evolve beyond rigid, handheld probes. Recent strides in materials science are now enabling this shift toward “soft” mechanics. For instance, Song et al. (2023) recently demonstrated that high‐performance piezoelectric ceramics (rare‐earth‐doped PMN‐PZT) can be successfully integrated into flexible PDMS substrates without sacrificing acoustic performance [93]. By achieving conformal skin contact, these wearable sensors can measure axial transmission parameters in real‐time, effectively bringing bone density assessment out of the radiology department and into daily life [93]. Although these metrics serve to complement rather than replace DXA, the ability to trend bone health through flexible, wearable ultrasound represents a significant leap forward in personalized medicine [92, 93].

### 3.3 Conditions of the Cardiovascular System

Cardiovascular diseases remain the leading cause of death worldwide and a major source of disability‐adjusted life years, accounting for a substantial share of global health loss [68]. Within this disease group, women and men diverge in mechanisms and presentation. Hormone‐dependent endothelial biology, microvascular regulation, immune–coagulation pathways, and life‐course exposures such as pregnancy, contraceptive use, and menopause alter risk and clinical phenotype in women [4, 94]. In ischemic heart disease, women more often exhibit coronary microvascular dysfunction and ischemia with non‐obstructive coronary arteries (INOCA), which is associated with symptom clusters that are less “typical” for obstructive epicardial disease (for example, dyspnea, fatigue, epigastric discomfort) and with a lower diagnostic yield of tests designed to detect fixed epicardial stenoses [95]. Heart failure with preserved ejection fraction shows a pronounced female predominance; the phenotype frequently includes diastolic dysfunction, concentric remodeling, arterial stiffness, and comorbid hypertension or obesity, presenting clinically with exertional dyspnea and exercise intolerance and requiring tailored diagnostic indices and management strategies [4]. Spontaneous coronary artery dissection disproportionately affects women, including during pregnancy and the postpartum period, and is a recognized cause of acute coronary syndromes in younger or middle‐aged women without traditional atherosclerotic risk factors [96]. Venous thromboembolism risk is likewise modulated by life‐course exposures, with pregnancy, the postpartum state, and use of estrogen‐containing contraception elevating thrombotic risk [94]. Taken together, these sex‐differences contribute to diagnostic delay and imperfect risk stratification, emphasizing the need for quantitative cardiovascular biomarkers (e.g., coronary flow reserve, diastolic filling dynamics, thrombus burden) and for targeted interventions. The sections that follow position ultrasound within these needs in women's cardiovascular health, with applications across ischemic heart disease, HFpEF, SCAD, and thrombosis.

#### 3.3.1 Cardiovascular Disorders in Women

**FIGURE 3:**
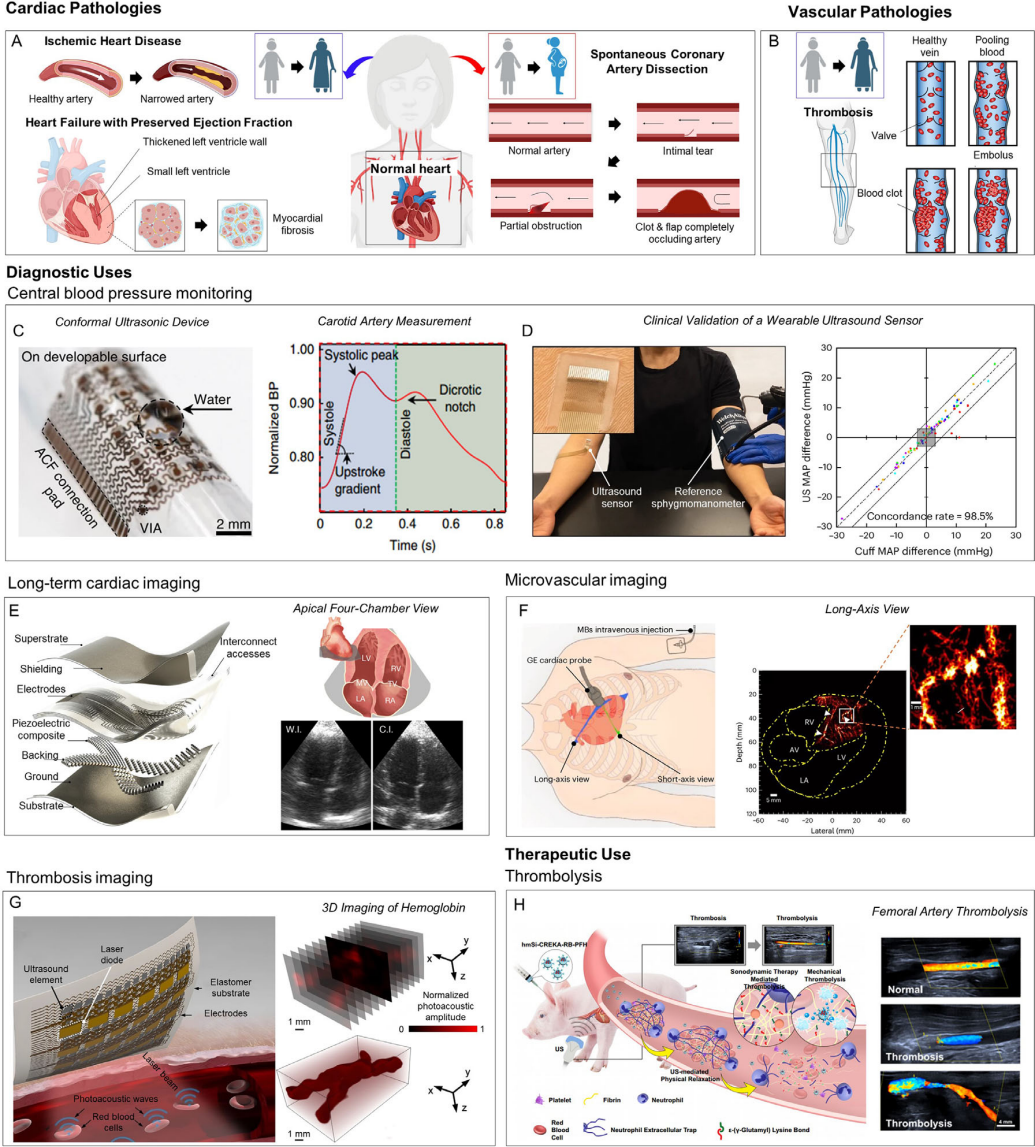
(A) Schematic of cardiac pathologies with female prevalence, which include ischemia with non‐obstructive coronary arteries (INOCA) and Heart Failure with Preserved Ejection Fraction (HFpEF) in post‐menopause populations and spontaneous coronary artery dissection (SCAD) in pregnant women. (B) Schematic of thrombosis, a vascular pathology with increased risk in women associated with age and use of estrogen‐containing medication. (C) Conformable ultrasonic device for the monitoring of central blood pressure (left) and its performance in measuring a typical waveform of the carotid artery (right), with annotations related to waveform features. (D) Clinical validation of a wearable ultrasonic sensor for blood pressure in comparison with a sphygmomanometer in the contralateral arm (left). Concordance rate of the Mean Arterial Pressure (MAP) between measurements during daily activities (right). (E) Schematic of a wearable cardiac ultrasonic imager (left) and B‐mode images of apical four‐chamber views, in comparison with commercial imagers. C.I., commercial imager; LA, left atrium; LV, left ventricle; MV, mitral valve; RA, right atrium; RV, right ventricle; TV, tricuspid valve; W.I., wearable imager. (F) Schematic depicting the ultrasonic probe position for cardiac imaging in the long‐ and short‐axis views of the heart and the injection of microbubbles (MBs) intravenously (left). Ultrasound localization microscopy (ULM) in the long‐axis view of the heart, illustrating deep microvasculature. AV, atrioventricular; LA, left atrium; LV, left ventricle; RV, right ventricle; (G) Schematic of a photoacoustic patch for 3D imaging of hemoglobin (left). Sequential slices of blood vessel (top‐right) and 3D imaging of hemoglobin in porcine tissue (bottom right). (H) Schematic of the theranostic platform for thrombi monitoring and thrombolysis. hmSi, hollow mesoporous silica; CREKA, Cys‐Arg‐Glu‐Lys‐Ala peptide; RB, Rose Bengal; PFH, perfluorohexane; US, ultrasound (left). Doppler US illustrating healthy condition, thrombosis, and thrombolysis in a femoral artery (right). Panels A, B: Created with Biorender.com and Microsoft PowerPoint. Panel C adapted with permission [97] 2018, Springer Nature. Panel D adapted with permission [98], 2024, Springer Nature. Panel E adapted under the terms of the CC‐BY License [99]. 2023, Springer Nature. Panel F adapted under the terms of the CC‐BY License [100]. 2024, Springer Nature. Panel G adapted adapted under the terms of the CC‐BY License [101]. 2022, Springer. Panel H adapted with permission [102]. 2024, Springer Nature).

Women are disproportionately affected by several cardiovascular diseases that present with sex‐specific mechanisms and clinical outcomes. Ischemic heart disease (IHD) is closely linked to menopause, as the decline in estrogen levels removes a key vascular protective factor. Estrogen normally promotes vasodilation, protects endothelial function, and maintains arterial elasticity; thus, its loss accelerates atherosclerosis and increases cardiovascular risk in postmenopausal women (Figure 3a). Similarly, heart failure with preserved ejection fraction (HFpEF) is highly prevalent among women, particularly after menopause, where hormonal changes contribute to increased arterial stiffness, abnormal ventricular relaxation, and impaired diastolic filling. Unlike other forms of heart failure, HFpEF often involves ventricular stiffening and myocardial fibrosis despite preserved systolic function. Another striking example is spontaneous coronary artery dissection (SCAD), a non‐atherosclerotic cause of acute coronary syndrome that occurs predominantly in younger women during pregnancy or the postpartum period. Hormonal and hemodynamic stresses associated with gestation and delivery are believed to weaken the arterial wall, predisposing to dissection. Finally, thrombosis represents a critical pathology in women, influenced by both hormonal fluctuations and lifestyle factors. Thrombi can form in multiple vascular territories, including deep veins of the legs, coronary arteries, or cerebral vessels, and may lead to embolism, stroke, or myocardial infarction (Figure 3b). This diversity underscores the systemic nature of thrombosis and its relevance to women's health.

#### 3.3.2 Central and Clinical Blood Pressure Monitoring

Blood pressure monitoring provides an essential window into cardiovascular risk, and its importance is amplified in female‐specific disease contexts. Central blood pressure, measured non‐invasively by advanced ultrasound devices, reflects the actual pressure load on the heart and proximal arteries (Figure 3c) [97]. The device operates in a pulse–echo ultrasonic mode, where conformal transducer arrays dynamically track the anterior and posterior vessel walls to reconstruct localized blood pressure waveforms with high spatial (∼0.4 mm) and temporal (∼500 µs) resolution. Targeting deep central vessels such as the carotid artery and jugular vein is clinically critical, as these sites directly reflect left‐ and right‐heart hemodynamics and provide more accurate prognostic information than peripheral measurements. The conformable architecture ensures stable coupling without excessive pressure, enabling reliable waveform acquisition even during motion. By resolving detailed waveform features such as the systolic peak, dicrotic notch, and diastolic phase, central pressure assessment provides a direct readout of ventricular–arterial coupling. Alterations in these features—such as an augmented late systolic peak or a diminished dicrotic notch—are indicative of increased arterial stiffness and impaired wave reflection. These hemodynamic changes represent hallmarks of postmenopausal vascular aging, contributing to elevated left ventricular afterload, diastolic dysfunction, and the subsequent progression of HfpEF and ischemic heart disease in women. Importantly, these wearable ultrasound sensors have undergone extensive validation across a wide range of clinical scenarios, from daily activities at home to outpatient visits, cardiac catheterization procedures, and intensive care monitoring (Figure 3d) [98]. In each setting, the devices showed close agreement with conventional methods, whether cuff‐based measurements or invasive arterial lines, while maintaining stable calibration over months of continuous use. Such robust validation highlights not only their accuracy in tracking both systolic and diastolic blood pressure but also their reliability during real‐life motion and physiological fluctuations, reinforcing their potential for early risk detection and long‐term management in women.

#### 3.3.3 Imaging Approaches for Long‐Term and Microvascular Monitoring

Although blood pressure measurement remains fundamental, it does not fully capture the complexity of cardiovascular disease in women. Long‐term cardiac imaging platforms provide extended assessment of chamber filling, ventricular emptying, and overall cardiac dynamics (Figure 3e) [99]. Long‐term imaging is enabled by the device's soft, stretchable substrate and liquid‐metal electrodes, which provide robust bonding and stable acoustic coupling even during body motion. An orthogonal piezocomposite transducer array delivers reliable biplane cardiac views without the need for manual probe rotation, while wide‐beam compounding and beamforming maintain image fidelity on curved chest surfaces. In addition, a non‐evaporating silicone coupling interface and low operating temperature ensure user comfort, supporting continuous monitoring from exercise to overnight use.

This is particularly relevant to HFpEF, where systolic function appears preserved but subtle impairments in ventricular relaxation and filling are often missed by spot checks. Continuous monitoring can reveal these transient diastolic abnormalities, which contribute to exercise intolerance and disease progression. Furthermore, advances in microvascular ultrasound now enable visualization of blood flow in small vessels and capillaries with unprecedented resolution (Figure 3f) [100]. Contrast‐enhanced ultrasound localization microscopy (ULM) accomplishes this by injecting microbubbles and localizing their trajectories across thousands of ultrafast frames, while motion correction and signal compounding stabilize the images to reconstruct detailed vascular maps and flow fields. Using standard transthoracic probes, ULM achieves ∼150–240 µm resolution and bedside quantification of microcirculatory hemodynamics without radiation. Clinically, this is especially valuable for detecting microvascular angina and SCAD, conditions that disproportionately affect women and are frequently missed by conventional angiography.

#### 3.3.4 Imaging Approaches for Long‐Term and Microvascular Monitoring

Photoacoustic thrombosis imaging provides 3D mapping of clot morphology and composition by combining optical excitation with acoustic detection (Figure 3g) [101]. In practice, pulsed laser light at 850 nm penetrates tissue and is preferentially absorbed by hemoglobin within thrombi, leading to thermoelastic expansion and the generation of ultrasound waves. These photoacoustic signals are then captured by integrated piezoelectric transducers and reconstructed into high‐resolution 3D images, enabling precise discrimination of thrombi from surrounding fat or muscle. Such capability allows accurate localization of venous clots in the legs, arterial thrombi in the heart, or embolic lesions in the brain. Beyond diagnosis, ultrasound‐mediated thrombolysis (Figure 3h) [102] has emerged as a powerful therapeutic modality. By combining focused ultrasound with targeted agents such as microbubbles, perfluorocarbon nanodroplets, or nanoparticle carriers, thrombi can be disrupted mechanically and lysed more effectively through cavitation and acoustic streaming. Newer theranostic platforms further incorporate sonosensitizers that generate reactive oxygen species upon ultrasound activation, breaking down neutrophil extracellular traps (NETs) and fibrin cross‐links that often render thrombi resistant to conventional tissue plasminogen activator (tPA) therapy. These approaches improve local blood flow, reduce the requirement for systemic thrombolytics, and are particularly promising for women at elevated risk of thromboembolism due to hormonal transitions, pregnancy, or prolonged immobility.

### 3.4 Conditions of the Nervous System

**FIGURE 4:**
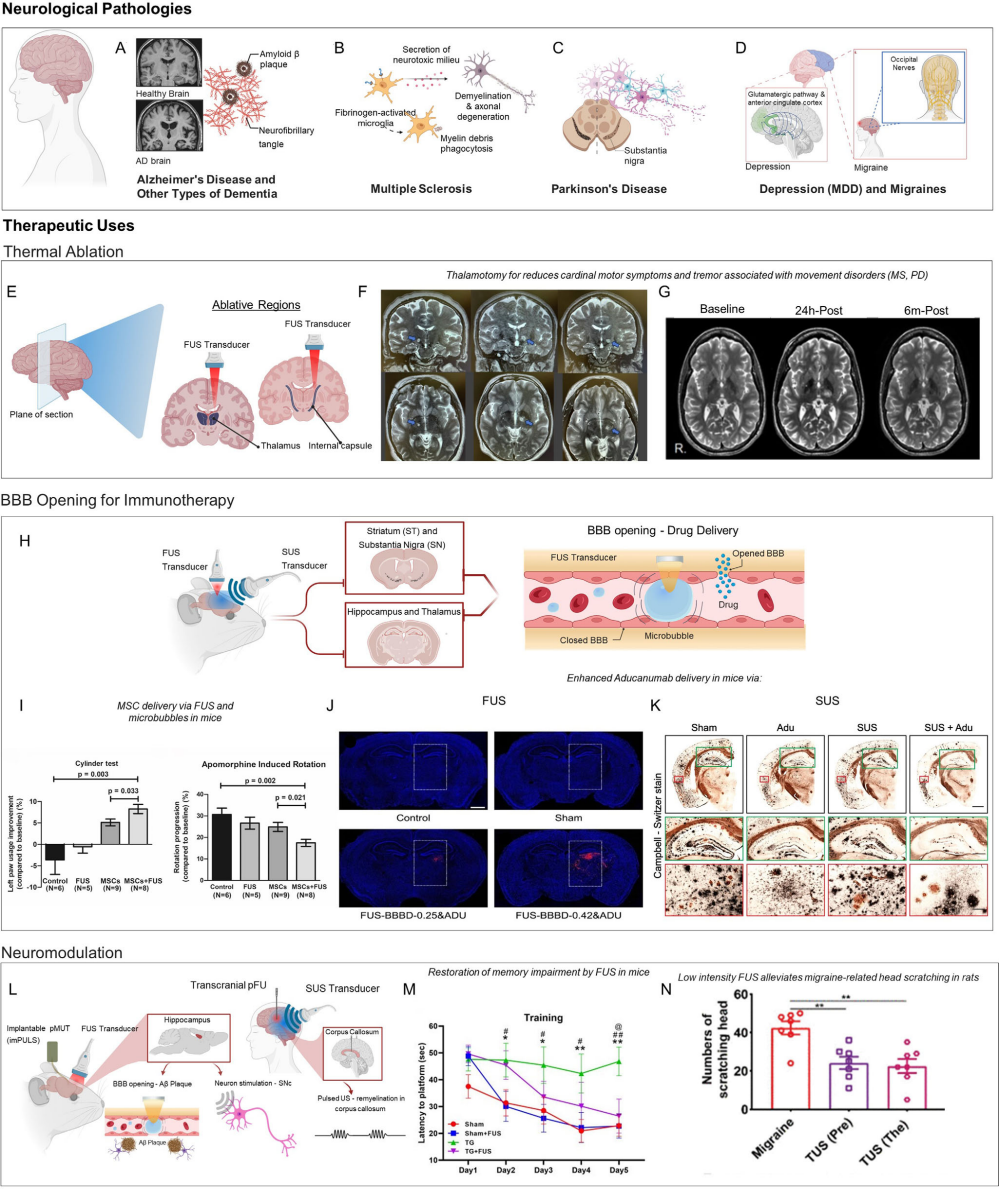
(A) Schematic of Alzheimer's disease and other dementias with MR images contrasting healthy and AD brains, which present amyloid‐β plaques and neurofibrillary tangles. (B) Schematic of multiple sclerosis pathobiology showing microglial activation and myelin loss leading to axonal degeneration. (C) Schematic of Parkinson's disease showing dopaminergic neuron loss in the substantia nigra. (D) Schematic of major depressive disorder highlighting anterior cingulate and glutamatergic pathways, alongside migraine with occipital nerve involvement. (E) Schematic of focused‐ultrasound ablation targets in the brain, showing the section plane and sonication paths to the thalamus and the internal capsule. (F) Immediate post‐procedure T2‐weighted MR images after unilateral subthalamotomy. Left, middle, and right panels show coronal (top) and axial (bottom) views from three representative cases. Blue arrows denote the subthalamic lesion, and the surrounding hyperintense rim indicates perilesional cytotoxic edema. (G) Axial T2 images at baseline, 24 h after left thalamotomy, and 6 months, demonstrating the focal lesion and absence of edema at 6 months. (H) Schematic of focused or scanning ultrasound with microbubbles to transiently open the blood–brain barrier in targeted regions (striatum/substantia nigra, hippocampus, thalamus), enabling delivery of immunotherapeutic agents. (I) Behavioral assessment shows left forelimb usage improvement in the cylinder test after MSCs+FUS treatment in a Parkinsonian model (left) and smaller progression on theapomorphine‐induced rotation test, indicating neurorestorative effects (right). MSCs+FUS group shows significant functional recovery compared to MSCs and control. MSCs = Mesenchymal Stem Cells. (J) Immunofluorescence showing aducanumab (ADU) delivery after focused‐ultrasound BBB opening with microbubbles. Control and sham (no effective acoustic pressure) show a negligible antibody signal. Sonication at 0.25 MPa yields focal intraparenchymal ADU, and 0.42 MPa produces broader uptake within the targeted region. Nuclei are DAPI (blue); ADU is Alexa Fluor 555 (red). (K) Campbell–Switzer silver staining of amyloid plaques in APP23 mice across four groups: Sham, aducanumab (Adu), scanning ultrasound (SUS), and SUS + Adu. Diffuse plaques appear black, and compact plaques amber. Top: whole hemisphere; middle: dorsal hippocampus (green inset); bottom: cortex overlying hippocampus (red inset). Plaque burden is lowest in the SUS + Adu group, consistent with ultrasound‐enhanced immunotherapy. Scale bar 1 mm. (**L**) Schematic of ultrasound neuromodulation techniques. Implantable pMUT and external FUS target the hippocampus to open the blood–brain barrier and stimulate neurons. Transcranial pFU and SUS apply pulsed ultrasound to the corpus callosum to promote remyelination. (M) Behavioral assay of spatial learning in mice across five training days. Four groups were tested: Sham, Sham+FUS, transgenic model (TG), and TG+FUS. The outcome is latency to reach a hidden platform, where lower values indicate better performance. Symbols mark significant group differences: ^*^ TG vs. Sham, # TG vs. Sham+FUS, @ TG vs. TG+FUS (*p* < 0.05 or *p*<0.01). (N) Behavior and cerebral blood flow (CBF) in the transcranial ultrasound stimulation (TUS) (Pre) [underwent TUS for 15 min, followed by a subcutaneous injection of nitroglycerin, which induces a migraine] and (TUS). The [subcutaneously injected with nitroglycerin, followed by TUS for 15 min] groups. The number of head scratches in migraine rats decreased significantly with ultrasound prevention and therapy (migraine group 42.5 ± 3.5, TUS (Pre) group: 24.1 ± 3.2, (TUS (The) group): 22.5 ± 3.6, mean ± SEM, N = 7 for each group, ^∗∗^ *p* < 0.01, Kruskal–Wallis test). However, no significant difference was observed between the ultrasound prevention and therapy groups. Panels A, B, C, D, E, H, L: Created with Biorender.com. Panel F adapted with permission [108] 2024, Springer Nature. Panel G adapted with permission [109] 2020, Sage Journals. Panel I adapted with permission [110] 2025, Springer Nature. Panel J adapted with permission [111] 2025, Springer Nature. Panel K adapted under the terms of the CC‐BY License [112] 2021, Springer Nature. Panel M adapted with permission [113] 2023, Elsevier. Panel N adapted under the terms of the CC‐BY License [] 2021, IEEE Publishing.

Neurological conditions constitute one of the largest contributors to worldwide health loss, representing the leading source of disability‐adjusted life years (DALYs) and the second leading cause of mortality [103]. Across this landscape, sex differences are evident: hormone‐dependent neurobiology, sex‐biased immune function, and chromosomal influences, together with life‐course events such as puberty, pregnancy, and menopause, shape how disorders present and progress in women [66, 104]. Within this context, the prevalence of Alzheimer's disease and overall dementia is two times bigger in women [105], and multiple sclerosis is diagnosed two to three times more frequently in women [106]. By contrast, Parkinson's disease is more common in men, but prevalence alone underestimates burden, as women with PD experience different clinical presentation and faster progression [107]. The following section highlights contemporary ultrasound platforms that can address the needs to improve women's brain health and their needs, with applications spanning Alzheimer's disease/dementia, Parkinson's disease, multiple sclerosis, major depressive disorder, and migraine (Figure 4a–d).

#### 3.4.1 Nervous Disorders in Women

Neuroscience research has revealed that men and women differ significantly in brain function and disease susceptibility, with sex‐linked factors influencing cognition, stress response, and the prevalence of neurological and psychiatric disorders [115]. These differences are particularly evident in Alzheimer's disease (AD) and other forms of dementia (ADOD), Multiple Sclerosis (MS), Parkinson's disease (PD), migraine, and depression. In AD, two‐thirds of clinically diagnosed cases of dementia and AD are women [116], with incidence differences becoming most apparent after 85 years of age. A pooled analysis of four population‐based prospective cohorts found that at 90 years of age, the incidence rate was 81.7 (95% CI, 63.8 to 104.7) in women compared with 24.0 (95% CI, 10.3 to 55.6) in men [117]. In MS, relapsing‐remitting MS (RR‐MS) is the most common form and occurs three times more frequently in females than in males [65], which is thought to result from more robust T cell‐mediated autoimmunity in females [65]. For PD, men are affected about twice as often as women, yet women experience distinctive symptoms, show different responses to pharmacological therapies and deep brain stimulation, and report lower quality of life compared with men [107]. According to the GBD 2021 worldwide data, rates of incidence, prevalence, and DALYs of migraine and depression are almost 2 times higher in women (Table 2).

A diverse array of ultrasound applications has shown promise for treating neurological conditions where sex dimorphisms play a role, particularly through thermal ablation, immunotherapy, and neuromodulation.

#### 3.4.2 Therapeutic Approaches Through Ultrasound‐Mediated Ablation and Drug Delivery

In thermal ablation, MR‐guided focused ultrasound (MRgFUS) thalamotomy markedly reduces refractory tremor present in multiple sclerosis and other movement disorders, with improvements in motor function and daily living activities lasting up to a year, offering a non‐invasive alternative to deep brain stimulation or radiofrequency [109, 118] (Figure 4e). In Parkinson's disease, MRgFUS subthalamotomy targeting the subthalamic nucleus leads to substantial motor improvements, with more than 50% reductions in tremor, rigidity, and bradykinesia at six months, while preserving balance and enhancing quality of life through precise thermal lesioning [108] (Figure 4f). Although thalamotomy carries risks of creating thalamic lesions with oedema, follow‐up studies show lesion size decreases and oedema resolves months after treatment [109] (Figure 4g).

Ultrasound can also facilitate immunotherapy through the transient opening of the blood–brain barrier (BBB) to facilitate delivery of therapeutics. This approach has successfully targeted human mesenchymal stem cells (MSCs) into the striatum and substantia nigra of Parkinsonian rats, producing significant behavioral improvements and protecting dopamine‐producing neurons compared with controls, highlighting its potential as a minimally invasive neuroprotective strategy [110] (Figure 4h,i). In Alzheimer's disease, MR‐guided focused ultrasound enhanced delivery of Aducanumab, a monoclonal antibody against amyloid‐β, achieving up to ∼60‐fold higher brain uptake without tissue damage [111] (Figure 4j). Similarly, scanning ultrasound (SUS) with microbubbles increased Aducanumab penetration by ∼5‐fold, and in combination with the antibody, achieved greater amyloid clearance and memory improvement than either treatment alone [112] (Figure 4k). For AD patients who are unable or unwilling to undergo anti‐amyloid‐β antibody treatment, drug‐free ultrasound approaches are of particular interest. Ultrasound alone, delivered through a wearable ultrasound system (WUS), was used in mouse studies to reduce amyloid‐β plaque burden while maintaining intensity levels below regulatory safety limits [119]. Such a miniaturized system, composed of elastomer‐encapsulated transducers driven via a flexible printed circuit and a small battery [119], exemplifies how ultrasound systems can become more portable in the transducer and driving units, enabling neurological treatments that are less‐resource intensive and less dependent on monoclonal antibody therapy.

#### 3.4.3 Ultrasound‐Mediated Neuromodulation

In recent years, ultrasound has emerged as a promising tool for neuromodulation, a field historically dominated by electrical or magnetic stimulation modalities. Acoustic vibrations can influence cell membrane properties through mechanical, thermal, and conformational changes, which can result in depolarization and the initiation of neural impulses [26]. Notably, ultrasound enables highly localized modulation of neural activity, even at low intensities, comparable to diagnostic levels. Current strategies for targeted acoustic delivery include transcranially focused ultrasound or minimally invasive implantables. FUS‐mediated BBB opening in Alzheimer's models improved cognition and increased LTP induction, restoring memory and spatial learning in the Morris water maze after six weeks of treatment [113] (Figure 4l,m). Repeated SUS at 1 MHz likewise enhanced spatial memory in APP23 mice without reducing amyloid‐β burden [120], with 1 MHz SUS‐treated mice showing fewer shocks and longer latency to first entry in the active place avoidance test (Figure 4l). Beyond non‐invasive approaches, implantable ultrasound devices expand the therapeutic scope. An implantable piezoelectric micromachined ultrasound transducer (pMUT), ImPULS, enabled spatially precise neuromodulation by stimulating dopaminergic neurons in the substantia nigra and modulating nigrostriatal dopamine release, representing a highly targeted tool with potential for Parkinson's therapy [35] (Figure 4l). In multiple sclerosis, transcranial pulsed focused ultrasound delivered in a patterned, low‐intensity protocol accelerated remyelination in the corpus callosum, confirmed by histological analysis, without tissue damage [121] (Figure 4l).

There are prominent sex differences in major depressive disorder (MDD), with women being twice as likely to be diagnosed as men and often experiencing greater symptom severity. Differences also persist in epidemiology, pathophysiology, and response to antidepressants, which highlights the need for alternative treatment approaches for MDD [122]. Focused ultrasound (FUS) has shown promise as an approach to treat MDD. Transcranial focused ultrasound (tFUS) increases brain‐derived neurotrophic factor (BDNF), a molecule linked to the pathogenesis of MDD, and promotes neurogenesis in mice [123]. Its capacity to open the blood–brain barrier (BBB) also facilitates BDNF transport into the brain. In humans, low‐intensity tFUS stimulation of the subgenual anterior cingulate cortex (sgACC) improved Montgomery–Åsberg Depression Rating Scale (MADRS) scores after six sessions [124]. MRI‐guided focused ultrasound (MRgFUS) capsulotomy of the internal capsule, a white matter tract linking the frontal cortex with deep brain structures and implicated in MDD and OCD, produced both targeted and widespread changes in neural activity [125]. However, MRgFUS capsulotomy faces limitations, including inconsistent lesioning success and limited response rates [126]. Low‐intensity focused ultrasound (LIFUS) targeting the medial prefrontal cortex has also been shown in rats to enhance synaptic plasticity in the VCA1–mPFC pathway and improve depression‐like behaviors [127].

Similar sex differences exist in migraine, which is two to three times more prevalent in women than in men. Women also experience longer migraine duration, slower recovery, and greater disability [128]. Pulsed ultrasound applied to the occipital nerve distribution in chronic migraine patients significantly reduced headache frequency, severity, and disability, with improvements sustained one and three months post‐treatment compared to sham control [129]. In a study linking head scratching as an expression of migraine symptomology and pathology (via a decrease in cerebral blood flow velocity) in rats, it was shown that low‐intensity transcranial FUS reduces head scratching without decreasing cerebral blood velocity [114]. This underscores ultrasound's ability to treat and prevent migraines in animals (Figure 4n), with an incredible translational potential.

### 3.5 Multisystem Conditions

**FIGURE 5:**
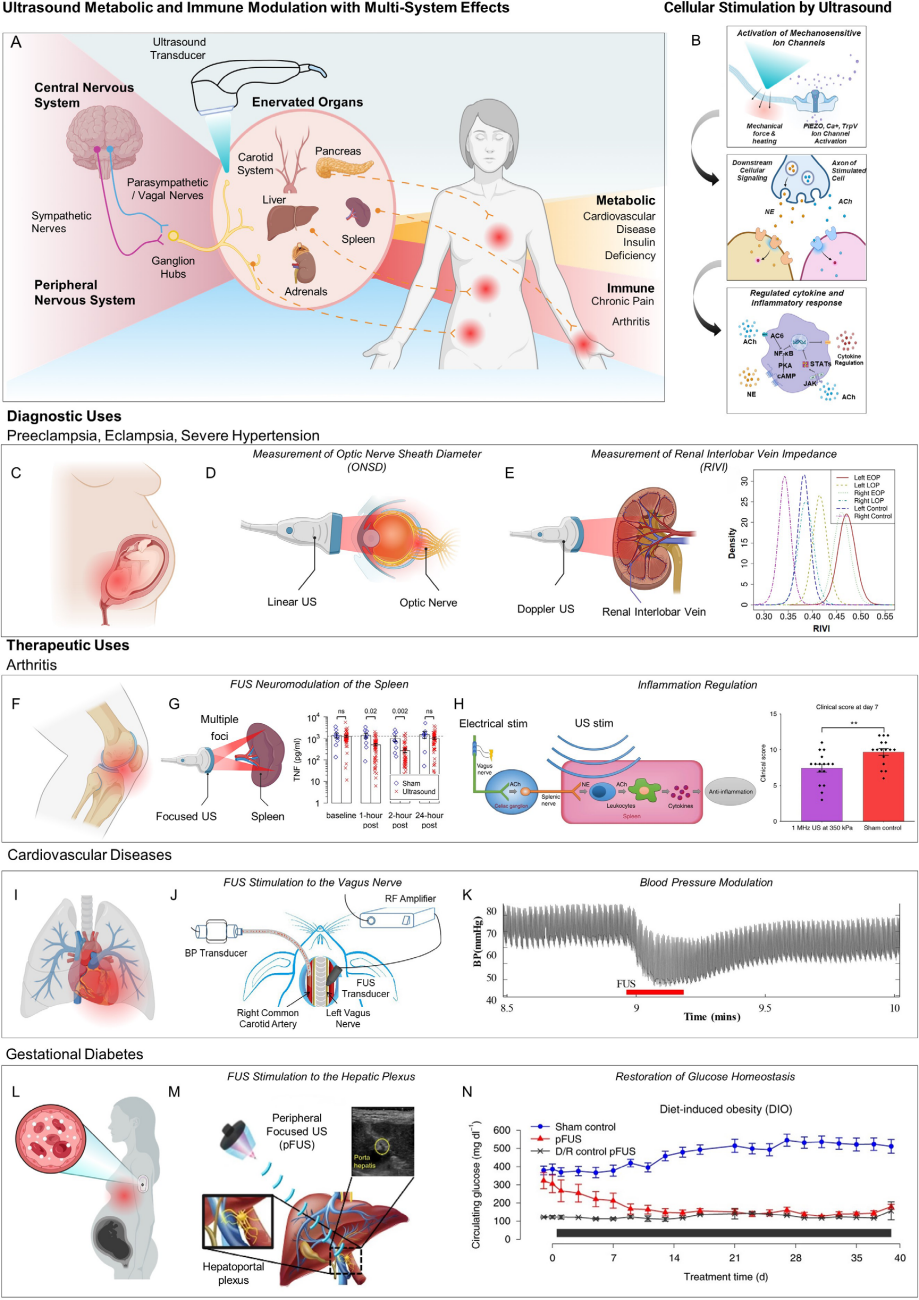
Ultrasound neuromodulation cascades to immunomodulation and metabolic stimulation, which treats multisystem conditions that involve immune, metabolic, autonomic, and endocrine pathways. (A) Organs in the body are enervated by the Peripheral Nerve System, which are critical points that can be targeted by ultrasound stimulation, for multi‐system metabolic and immune therapy. (B) Effects are initiated by mechanosensitive ion channels activation in neurons that produce downstream signaling to immune cells via Acetylcholine (ACh) and Norepinephrine (NE). These signals help immune cells to normalize inflammatory pathways via cytokine downregulation via the Janus kinase‐signal transducer and activator of transcription (JAK‐STAT) pathway and inhibition of interleukin‐6 (IL‐6) transcription via nuclear factor‐kappaB (NF‐kB) transcriptional activity caused by effects on the cyclic adenosine monophosphate‐dependent (cAMP‐dependent) pathway and adenylyl cyclase 6 (AC6). (C) Multi‐system diagnostic use case where measurement of Optic Nerve Sheath Diameter (ONSD) in eyes (D) and Renal Interlobar Vein Impedance (RIVI) in kidneys (E) can be indicators of preeclampsia and acutely high blood pressure. EOP, early‐onset preeclampsia; LOP, late‐onset preeclampsia. (F) Chronic inflammatory conditions such as arthritis can be treated with focused ultrasound stimulation of the spleen and splenic nerve. In humans (G) and in mice (H), stimulation has been shown to reduce biomarkers such as tumor necrosis factor (TNF) for 24 h and improve clinical scores across chronic periods. (I) Cardiovascular diseases are rooted in early conditions and symptoms relating to elevated blood pressure. Focused ultrasound stimulation of the vagus nerve in mice (J) has shown to lower blood pressure under constant stimulation (K). (L) Diabetes uniquely affects women by worsening at‐risk complications in reproductive and cardiovascular health. Focused ultrasound stimulation of the hepatoportal plexus in the liver of mice (M) has shown a significant reduction in circulating glucose (N) over a month‐long period. Panels A, B, C, D, E, F, G, I, L: Created entirely or partially with Biorender.com. [] 2012, Wolters Kluwer Health, Inc. Panel E adapted with permission [136] 2020, John Wiley and Sons. Panel G adapted with permission [138] 2023, Elsevier. Panel H adapted under the terms of the CC‐BY License [139] 2019, Springer Nature. Panels J and K adapted under the terms of the CC‐BY License [140]. 2020, Frontiers Media SA. Panels M and N adapted with permission [131]. 2022, Springer Nature.

Multisystem conditions that couple immune, metabolic, autonomic, and endocrine pathways account for a large share of global health loss, combining long‐term disability with meaningful mortality [68]. In this broad category, sex differences arise from hormone‐dependent physiology, immune dimorphism, and chromosomal factors; pregnancy, contraceptive exposure, and menopause further shape inflammatory set‐points, metabolic control, hemodynamics, and stress responses in women [4, 66, 130]. This heterogeneity creates diagnostic gaps and underscores the need for sex‐specific, quantitative biomarkers of systemic state, such as inflammatory activity, hepatoportal glucose sensing, baroreflex sensitivity, and stress‐hormone dynamics, and for targeted interventions. In this context, organ hubs with system‐level leverage become actionable entry points that ultrasound can access for targeted diagnostics and therapy: the spleen as a gateway to the inflammatory reflex, the liver and portal nerve plexus as nodes for autonomic regulation of glucose homeostasis, the carotid baroreceptor as a sensor for blood‐pressure reflexes, and the adrenal glands as effectors of catecholamine‐mediated stress responses [4, 66, 131, 132] (Figure 5a,b). Subsequent sections outline how contemporary ultrasound platforms align with these needs in women's health, focusing on the spleen, liver, carotid baroreceptor, and adrenal glands.

#### 3.5.1 Diagnostic Ultrasound in Multisystem Conditions

Diagnostic ultrasound has traditionally been valued for its noninvasive imaging capabilities, but an emerging body of evidence demonstrates its potential to quantify physiologic biomarkers that reflect the state of interconnected organ systems. This multi‐system perspective is particularly relevant in women's health, where conditions such as preeclampsia exemplify the convergence of vascular, renal, and neurological dysfunction.

One salient example is the measurement of optic nerve sheath diameter (ONSD) as a surrogate for intracranial pressure (ICP). Transorbital ultrasound enables bedside quantification of ONSD, which expands in response to elevated ICP due to direct communication between the subarachnoid space and the optic nerve sheath. In women with preeclampsia (Figure 5c), ONSD values are significantly greater than in healthy pregnant controls, with thresholds around 5.8 mm demonstrating high specificity (≈97%) and good sensitivity (≈85%) for the detection of cerebral edema and raised ICP (Figure 5d) [133, 134]. Such findings underscore that a single ocular biomarker can act as a window into central nervous system integrity, offering clinicians a noninvasive means to anticipate neurologic complications in hypertensive pregnancy disorders [135].

Similarly, Doppler interrogation of the intrarenal vasculature extends the diagnostic reach of ultrasound beyond structural imaging, providing insights into systemic vascular and renal hemodynamics. While arterial resistive indices show limited discriminative value, venous parameters such as the renal interlobar vein impedance index (RIVI) have emerged as promising biomarkers (Figure 5e). Elevated RIVI values are strongly associated with preeclampsia, with some studies reporting odds ratios exceeding 17 for predicting disease onset [136, 137]. These indices capture venous congestion and compliance abnormalities that are not confined to the kidneys but rather reflect systemic vascular dysfunction characteristic of preeclampsia.

Together, these examples illustrate how ultrasound biomarkers can reveal multi‐system derangements through focused, organ‐specific measurements. In preeclampsia, assessing ONSD links ocular findings to cerebral pathophysiology, while renal venous Doppler indices connect kidney flow dynamics to systemic vascular health. Such approaches demonstrate the potential of diagnostic ultrasound to act not only as a window into discrete organs, but also as an integrative tool for monitoring the interplay of neurologic, renal, and cardiovascular systems in women's health.

#### 3.5.2 Therapeutic Ultrasound in Multisystem Conditions

Therapeutic ultrasound stimulation has emerged as a novel strategy to modulate neural circuits along the pathways that innervate peripheral organs. Unlike traditional diagnostic ultrasound, which is designed to image tissue, therapeutic focused ultrasound delivers mechanical energy that can activate mechanosensitive ion channels in nerve fibers, depolarize local neural endings, and thereby engage organ‐specific reflexes. These effects extend to both neuromodulation, by altering the activity of autonomic pathways, and immunomodulation, by rebalancing inflammatory cytokine signaling through neuroimmune reflexes such as the vagus–spleen axis (Figure 5b).

This approach offers several distinct advantages over conventional therapies. First, it is non‐invasive: low‐intensity focused ultrasound can be delivered externally without surgical implants, enabling repeated treatments without procedural risk. Second, it is non‐pharmacological, avoiding the systemic side effects, tolerance, and drug–drug interactions common to immunosuppressants, antihypertensives, or analgesics. Third, it is inherently personalizable: stimulation can be titrated to individual physiological responses, adjusted to disease states, and potentially combined with imaging guidance to target specific organ regions or nerve plexuses.

By leveraging the body's own regulatory circuits rather than overriding them with drugs, therapeutic ultrasound neuromodulation has the potential to provide safe, adaptive, and durable treatment options for chronic and undertreated diseases. In the context of women's health—where autoimmune disorders, metabolic dysregulation, cardiovascular disease, and chronic pain are prevalent yet often undertreated—this organ‐directed, circuit‐based approach opens new avenues for precision medicine.

Autoimmune diseases such as rheumatoid arthritis (Figure 5f) and systemic lupus erythematosus affect women at rates two to ten times higher than men and are often undertreated due to diagnostic delays and limited therapeutic options [66, 141, 142]. These disorders are marked by chronic inflammation and excess production of cytokines such as TNF‐α, IL‐1β, and IL‐6. Focused ultrasound stimulation of the spleen [138] (Figure 5g) activates the cholinergic anti‐inflammatory pathway, in which norepinephrine release from splenic nerves induces acetylcholine secretion by specialized T cells, suppressing macrophage cytokine release. Preclinical studies demonstrate that spleen‐targeted ultrasound reduces cytokine surges and ameliorates arthritis severity, with effects comparable to invasive vagus nerve stimulation [138, 139] (Figure 5h). For women disproportionately affected by autoimmune arthritis, spleen ultrasound provides a noninvasive, drug‐sparing option for restoring immune balance.

While immune dysregulation drives much of women's autoimmune disease burden, metabolic disorders such as polycystic ovary syndrome (PCOS) and gestational diabetes (Figure 5l) also hinge on disrupted signaling between peripheral organs and the central nervous system. Ultrasound neuromodulation of the liver and adrenal glands offers a new axis of intervention. The liver, particularly its portal plexus, is a central hub for glucose sensing and insulin regulation. Ultrasound stimulation of hepatic nerves (Figure 5m) has been shown to restore glucose homeostasis in rodent and swine models of type 2 diabetes, improving insulin sensitivity and lowering blood glucose [131] (Figure 5n). This neuroimmune mechanism suppresses systemic inflammation and supports metabolic balance. For women, in whom hormonal cycles, PCOS, and pregnancy uniquely shape metabolic risk, noninvasive modulation of the liver circuits could directly address both inflammatory and metabolic components of disease.

As metabolic health is intimately linked to vascular health, another pressing need in women's medicine lies in cardiovascular disease (Figure 5i). Here, neuromodulation of carotid baroreceptors emerges as a promising intervention. Cardiovascular disease is the leading cause of death in women, yet remains underdiagnosed and undertreated due to atypical symptom presentation and a higher prevalence of microvascular dysfunction. Focused ultrasound applied to the carotid sinus or cervical vagus nerve has been shown in animal models to modulate baroreceptor reflexes, lowering blood pressure and heart rate in a dose‐dependent fashion [140] (Figure 5j,k). For women with resistant hypertension or autonomic imbalance, ultrasound‐based carotid stimulation could offer a precision alternative to systemic antihypertensives, providing noninvasive modulation of cardiovascular reflexes tailored to female pathophysiology.

In addition to immune, metabolic, and cardiovascular disease, women disproportionately suffer from chronic pain conditions such as fibromyalgia, which are strongly linked to neuroimmune dysregulation. Fibromyalgia and other chronic pain syndromes affect women up to nine times more frequently than men and are characterized by central sensitization, glial activation, and chronic cytokine imbalance. Ultrasound neuromodulation can directly excite mechanosensitive ion channels such as Piezo and TRP family receptors on neurons, thereby modulating pain signaling pathways. Rodent studies show reduced neuroinflammatory markers and altered pain thresholds after ultrasound neuromodulation, while human transcranial ultrasound studies have demonstrated modulation of cortical excitability linked to sensory perception [143]. For women with chronic pain who often face dismissal or limited pharmacologic options, ultrasound provides a mechanistically grounded, non‐pharmacological alternative capable of rebalancing neuroimmune function.

## 4 Concluding Remarks and Future Perspectives

### 4.1 Outlook

**FIGURE 6:**
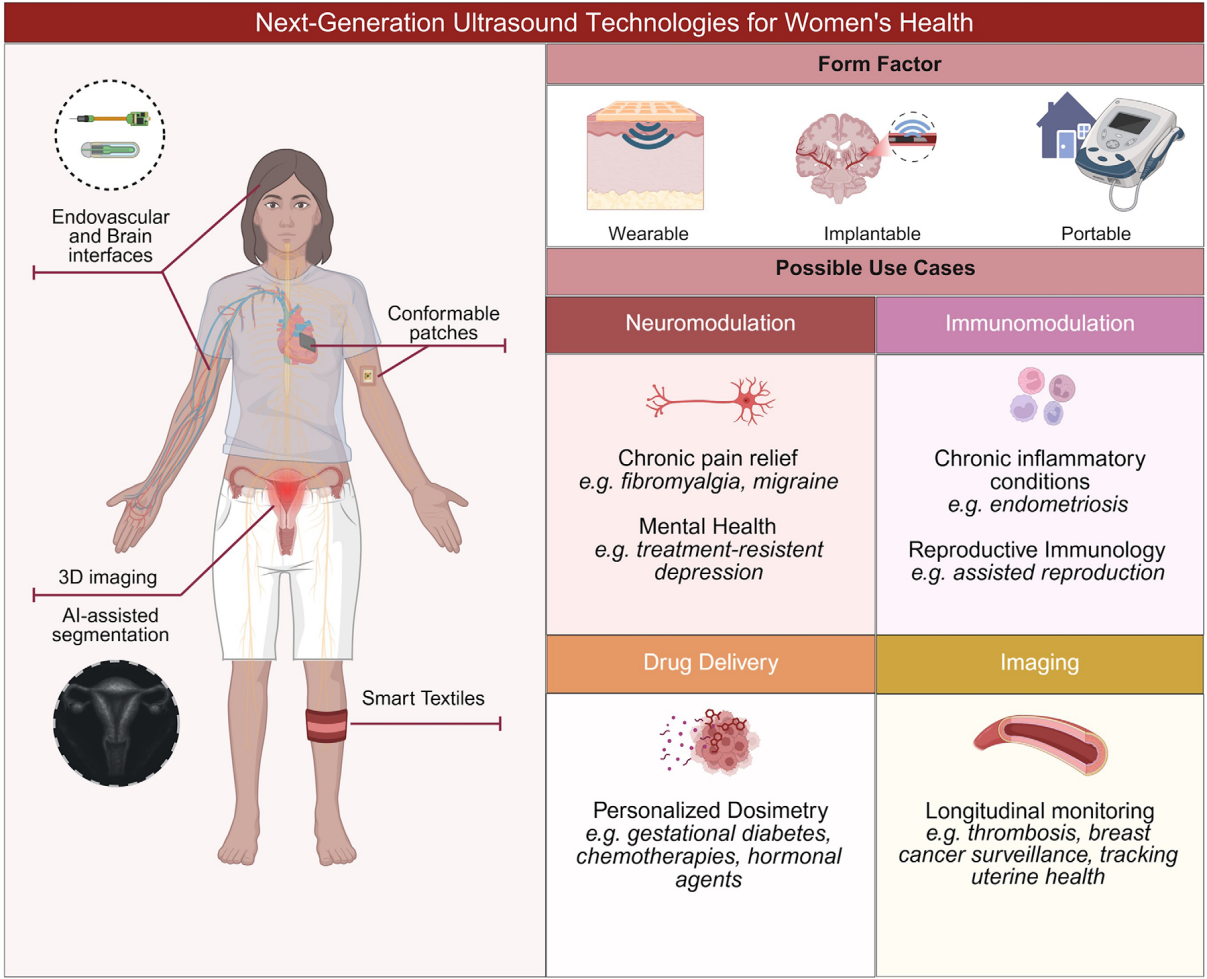
Emerging ultrasound technologies offer a range of wearable, implantable, and portable form factors designed to interface with the human body, especially relevant for women's health conditions. Illustrated use cases highlight the anatomical diversity and clinical relevance of these ultrasound‐based platforms in the future. Figure created with BioRender.com.

Ultrasound technology is well‐positioned for transformative growth in women's health, not only because of the plethora of applications that it covers, but also the ongoing miniaturization of transducers and electronics, which translates into portable, handheld, or implantable ultrasound devices, and the advent of flexible and stretchable transducers, which enable wearable devices that provides real‐time information [144]. Conventional ultrasound transducers are rigid and bulky, mainly because their main components are a piezoelectric material layer, for the conversion of electrical energy into mechanical vibration, and additional backing and matching layers, for dampening of unwanted signals and minimizing acoustic impedance mismatch, respectively. To realize thin, compact devices, strategies such as micromachined ultrasound transducers (MUTs), primarily capacitive (CMUTs) and piezoelectric (PMUTs) [145], improvements in manufacturing methods such as 3D printed, acoustic metamaterials [146], and miniaturization of electronic components for signal processing and power management have been deployed. The next generation of ultrasound devices will be able to adapt to the complex and variable female anatomy (e.g., breast and uterus anatomy, gravid abdomen) through non‐invasive or minimally invasive interfaces, conformable substrates, or integration into smart textiles (Figure 6). Emergent wearable devices that maintain stable performance and adhesion on strongly curved surfaces [57] or even have their focus spatially tuned according to the tissue deformation [147] are examples of devices that advance toward a truly body‐conformable therapy. Such flexibility offers unprecedented opportunities for outpatient care or at‐home treatment, which can address gaps in chronic pain management (e.g., fibromyalgia, migraine), mental health treatment (e.g., treatment‐resistant depression), and address chronic inflammatory conditions (e.g., endometriosis).

Leveraging these novel interfaces, personalized dosimetry, where ultrasound parameters are fine‐tuned for individual needs, could revolutionize the treatment of conditions such as gestational diabetes, hormone‐responsive disorders, and various (uterine, ovary, breast) cancers. Within immunomodulation and neuromodulation, suborgan electrical stimulation of the vagal‐adrenal axis can modulate catecholamine and dopamine release [148] and optogenetic stimulation of the pancreas has been shown to modulate insulin and B Cell proliferation [149], presenting major future opportunities for ultrasound to also be used to explore suborgan stimulation.

Furthermore, ultrasound's ability to support longitudinal monitoring of thrombosis [150], breast cancer recurrence [36], uterine/ovarian health [151], among others, opens the door to continuous, personalized care outside the clinical setting. Portable and wearable ultrasound devices empower patients and providers with real‐time tracking of disease progression or treatment response, particularly valuable in managing chronic and complex conditions. Importantly, for ultrasound systems to become fully operator‐independent for long‐term use, wearable transducers are not sufficient: data processing units need to be wireless, and the measurements need to be reliable even with moving targets. Such requirements have been proved feasible to be implemented, and continuous physiological monitoring of cardiac output with a fully integrated ultrasound system has been recently reported [152]. Integration with AI and digital health platforms further augments diagnostic accuracy and supports individualized treatment adjustments [153]. The maturity of ultrasound as an imaging modality, combined with the abundance of biomedical imaging datasets, provides a strong foundation for developing robust, device‐integrated intelligent architectures capable of supporting both ethical use and broader accessibility.

Such co‐innovation in device miniaturization, power electronics, soft interfaces, and advanced, safe packaging fit for consumer use — combined with AI‐augmented processing and interpretation—has the potential to expand ultrasound from a primarily clinical tool to one that also supports continuous personal health and wellness. This is especially compelling for women's health, where diverse physiological presentations, variable symptom reporting, and long‐standing inequities in diagnostic attention have historically complicated timely and accurate care. Integrating intelligent, user‐friendly ultrasound technologies directly into everyday health practices could help bridge these gaps and enable more responsive, individualized monitoring.

These advances align strongly with the broader healthcare shift toward decentralized, patient‐centered models that prioritize accessibility, convenience, and ongoing engagement, particularly for women's health needs that demand nuanced and adaptive care.

### 4.2 Challenges

#### 4.2.1 Wearable Ultrasound Limitations

While women report that using a wearable device provided valuable information about their own bodies, either validating their perceptions of physiological alterations or allowing them to uncover new insights, they also describe that wearing self‐tracking equipment created bodily discomfort, frustration when it malfunctioned and a sense of insecurity when the messages presented were different from the ones provided by a physician [154]. Wearable ultrasound technologies face additional hurdles, as they still lack standardized evaluation and comparison, due to the wide parameter space (e.g., frequency, acoustic intensities, focusing area), testing setups, and application scenarios [30, 144]. In addition, for the promise of operator‐independent ultrasound to be realized, further optimization of soft, biocompatible coupling media is needed to balance long‑term skin wearability with stable acoustic coupling and accurate beamforming [30, 144].

Beyond challenges in hardware comfort and biosignal data fidelity, concerns about data privacy permeate the use of consumer wearables in health research, as companies operate under little oversight in relation to data collection, storage, and aggregation, especially when for‐profit organizations might have competing interests with consumer users [155]. These concerns are especially exacerbated when related to women's sensitive information, such as menstrual cycles and broader reproductive health information, which can be misused to deepen discrimination in contexts such as employment insurance and healthcare access [156]. For wearable ultrasound and other health technologies to truly empower female health, it is imperative that their implementation rests on robust frameworks for data privacy and explicitly defined pathways for integrating user data with clinical practice, ensuring the benefits enhance, not undermine, physician‐patient relationships.

#### 4.2.2 Safety and Dosimetry in Personalized Therapeutic Ultrasound

Especially related to therapeutic ultrasound, the absence of standardized dosimetric measures can result in substantial variability in actual delivered dose, risk profile, and efficacy from one device to another [157]. This exposes patients to the risk of both under‐ and overtreatment, hinders effective quality assurance, and slows widespread clinical use of promising therapies. While there is no universal metric that can singlehandedly predict all possible ultrasound‐based biological effects, methods to monitor heating include the quantification of the thermal dose through Cumulative Equivalent Minutes at 43°C (CEM 43), covered in Note S1, that can be quantified through thermocouples in vitro or MRI thermometry maps in vivo [157, 158]. Cavitation can be detected by broadband emissions capture in vitro with hydrophones and in vivo with an additional ultrasound receiver [157, 158], and can be predicted by the Mechanical Index metric (Note S1). Thermal and cavitation limits are therefore informed by not only the intended application of US but also the type of organ or tissue and the presence of cavitation nuclei [159]. These monitoring methods remain recommendations or consensus of research committees, such as the American Institute of Ultrasound in Medicine (AIUM) [158], instead of mandates from the FDA, which exist for the marketing of commercial ultrasound used for diagnostic purposes [52]. Therefore, there is still a need for standardization of both monitoring technologies and exposure limits for the growing indications of therapeutic ultrasound.

Adverse effects vary by tissue intended use. In a study of clinical use of MRgHIFU in China for treatment of benign and malignant tissues, adverse effects include skin burns (22.99%), edema, pain, and rare severe events like tumor rupture, embolism, or death [159, 160]. Long‐term risks may involve fibrosis or vascular changes, underscoring the need for surveillance [158, 159]. To improve the safety of patients undergoing therapies utilizing US, establishing universally accepted dosimetric protocols, including validated measurement techniques, anatomical modeling, and reference standards, is crucial. These standards will enable accurate dose‐response assessments, greater patient safety, and a more effective basis for regulatory approval and global clinical deployment.

#### 4.2.3 AI‐Assisted Diagnostics

While artificial intelligence (AI) holds tremendous promise for enhancing precision medicine by identifying medically relevant patterns and supporting diagnostic decisions, sex and gender differences are often overlooked during AI model development, resulting in algorithms that inadequately capture female‐specific disease manifestations and pathophysiology [161]. Historical underrepresentation of women in clinical trials and biomedical datasets leads AI models to inherit male‐centric data biases, increasing risks of misdiagnosis and suboptimal care for women.

Recent studies show that AI diagnostic tools can exhibit reduced accuracy and fairness across sex, gender, and ethnic dimensions, sometimes relying on demographic assumptions that amplify inequities [162, 163]. Such biases are complicated by limited interpretability and transparency in AI systems, making it challenging to detect and correct discriminatory patterns [163]. To mitigate these issues, incorporating sex‐ and gender‐specific performance metrics, expanding the inclusion of diverse female populations in training datasets, and fostering interdisciplinary collaborations spanning clinicians, AI developers, and impacted patient communities is necessary [161]. Without deliberate interventions, AI risks digitizing and amplifying entrenched healthcare disparities rather than advancing equitable outcomes for women's health. Addressing these gaps demands both the intentional inclusion of diverse, representative data and the development of explicit strategies for bias evaluation and correction throughout the AI pipeline.

Beyond challenges in overcoming training bias, the clinical adoption of AI raises ethical and legal uncertainties. Questions remain regarding physician accountability when diagnostic errors arise from AI‐assisted decisions, which lack nuance and contextual information, such as family history, age, among other factors [164]. These emerging issues underscore the need for frameworks that clarify liability and ensure that fairness and transparency guide AI integration into healthcare practice.

#### 4.2.4 Clinical Endpoints

Developing reliable sex‐ and condition‐specific endpoints remains critical to capturing meaningful clinical responses and guiding treatment. Current clinical trials and device evaluations often overlook sex as a biological variable, limiting the precision of outcome measures and potentially masking important differences in how diseases manifest and respond to treatments between women and men. Regulatory bodies such as the FDA have issued guidance emphasizing the need for thorough sex‐based data analysis to ensure that endpoints adequately reflect differential safety and effectiveness across sexes, aiding in more accurate categorization of responders and non‐responders.

Sex‐specific endpoints are especially important in conditions with known divergent pathophysiology or treatment effects, where standardized outcome measures tailored to female biology can improve risk assessment, optimize dosing, and reduce adverse effects. Furthermore, the identification and validation of sex‐specific genomic and molecular biomarkers linked to clinical endpoints enable more nuanced and individualized prognostic and therapeutic decisions, replacing outdated one‐size‐fits‐all paradigms. Overall, integrating sex‐ and condition‐specific endpoints into clinical trial design and routine practice is vital to advancing equitable and effective personalized medicine for all patients.

#### 4.2.5 Inequalities in the Adoption of Ultrasound

Despite its potential, global access to ultrasound remains uneven. A survey of healthcare providers in LMICs found that the most commonly cited obstacles to ultrasound use include limited availability of equipment, shortages of trained personnel, and competition for shared devices [165]. Even when clinicians recognize the clinical value of US examinations, logistical constraints often prevent consistent use. Only one‐third of surveyed facilities reported having institutional policies or guidelines for ultrasound usage, underscoring the lack of standardization in practice and governance [165].

Disparities in access are not confined to low‐income settings. A study in Saskatchewan, Canada revealed persistent sociodemographic and geographic inequities in ultrasound utilization [166]. Patients in rural or remote communities, individuals with lower socioeconomic status, and Indigenous populations are less likely to receive recommended prenatal imaging compared to urban or wealthier groups. Factors such as geographic isolation, younger maternal age, lower education levels, multiparity, and substance use further compound these disparities in prenatal care uptake [166].

Access to ultrasound can further be complicated by state and local policy. For example, laws enacted to prevent prenatal sex selection (such as India's PC‐PNDT [167] framework) serve an essential public‐health purpose but have also created substantial regulatory burdens and, at times, punitive enforcement practices that inadvertently restrict access to obstetric and general diagnostic ultrasound, particularly in smaller or rural facilities. Emerging “smart” ultrasound systems can offer a complementary path forward by embedding safeguards against misuse directly into the technology itself (e.g., automated detection of prohibited scanning patterns or real‐time shutdown triggers), thereby reducing reliance on workforce reforms or policy shifts that may take decades to materialize.

Even in specialized populations, the pattern persists. A national survey across U.S. Veterans Affairs medical centers showed limited adoption of point‐of‐care ultrasound in geriatric clinics (only 15% reported routine use), primarily due to equipment shortages and inadequate training [168]. Taken together, these findings illustrate that while ultrasound holds vast potential to advance equity in healthcare delivery, its benefits remain stratified along lines of geography, socioeconomic status, and institutional capacity. Bridging these gaps will require coordinated efforts in training, policy development, and resource allocation to realize ultrasound's full potential as a global diagnostic equalizer.

## 5 Conclusion

In this review, we highlight how ultrasound technologies are well‐positioned to address gaps in medical research of diseases and conditions that affect women preferentially and differentially. Besides conditions of the Reproductive System, we show evidence that diseases in the Cardiovascular, Nervous, and Multi‐System present different prevalence and phenotypes in women, demanding personalized and longitudinal monitoring, and treatment platforms that are versatile and can have adjustable dosage. Enabling diagnosis and treatment at home or outside of hospital settings can also be beneficial in order to address systemic barriers to care that women face. Ultrasound technologies are capable of offering diagnostic information such as real‐time anatomical and functional assessment, blood flow monitoring, and tissue characterization, while also supporting therapeutic applications ranging from targeted ablation to neuromodulation and drug delivery. The continued miniaturization and development of wearable, implantable, and point‐of‐care ultrasound devices hold particular promise for advancing decentralized and patient‐centered care in women's health. However, future progress requires addressing persistent challenges in safety standards, personalized dosimetry, equitable AI integration, and the creation of sex‐specific clinical endpoints. By confronting these limitations while leveraging technological advances, ultrasound has the potential to transform the landscape of women's healthcare, providing safer, more accessible, and more precise modalities that improve outcomes and quality of life for women worldwide.

## Funding

National Science Foundation CAREER: Conformable Piezoelectrics for Soft Tissue Imaging (grant no. 2044688), National Science Foundation Award (grant no. 2524831), Acıbadem Mehmet Ali Aydınlar University School of Medicine Merit‐Based Scholarship Program.

## Conflicts of Interest

The authors declare no conflicts of interest.

## Supporting information




**Supporting File**: adma72366‐sup‐0001‐SuppMat.docx.

## Data Availability

The authors have nothing to report.
