# Supplementary material for: Ultrasound in Women's Health: Mechanisms, Applications, and Emerging Opportunities
Source: Adv Mater. 2026 Feb 5;38(14):e20454. doi: 10.1002/adma.202520454 (PMC12966983; doi:10.1002/adma.202520454)
Supplement: Supplementary file 1 — Supporting File: adma72366‐sup‐0001‐SuppMat.docx. [file ADMA-38-e20454-s001.docx]

Supplementary Information

**Ultrasound in Women's Health: Mechanisms, Applications, and Emerging Opportunities**

*Sarah B. Ornellas, Bilal Kizilaslan, Aastha Shah, Jason F. Hou, Yoonsoo Shin, Alejandra Hernandez Moyers, Claudia Lozano, and Canan Dagdeviren**

**Supplementary Note S1**

Glossary

**Acoustic impedance (Z):** The resistance presented by the medium to wave propagation. It can be expressed by the equation:

$$Z= \rho*c$$

where ρ represents the density of the medium and c is the speed of sound in the medium.

**Amplitude (A):** The peak pressure (i.e. the ‘height’) of the wave, which are fluctuations above and below the ambient pressure. Its measurement units are Pascal.

**Boundary width:** The lateral width of the boundary is a key factor in determining the strength and type of reflection back towards the transducer. The boundaries can be defined as:

Type 1: Boundary width ~10λ: Such an interface behaves as a perfect specular reflector, and the wave follows Snell’s law of reflection based on the angle of incidence of the wave

Type 2: Boundary width ~λ: Some of the energy is scattered isotropically, while a part of it makes it back to the source

Type 3: Boundary width < 0.1λ: The energy is scattered uniformly following Rayleigh scattering, and only a very small portion of the energy (on the order of (D/λ)^4 makes it back to the source

**CEM43 ^o^C:** A thermal dose metric, which accounts for the cumulative equivalent minutes at the reference temperature of 43°C. It quantifies tissue exposure to heat and can be used to predict tissue damage [1]. The formula below can be used to convert any time period/temperature combination into the standard CEM43^o^C:

$$CEM43℃=\sum_{i}^{n} t_{i}\cdot R^{(43-T_{i})}$$

Where t_i_ is the time period duration in minutes, T_i_ is the average temperature during that period, and R is a compensation factor for ranges of temperature (for T_i_ < or at 43^o^C, it can be set to 0.25 and for T_i_ >43^o^C, it can be set as 0.5).

**Frequency (f):** The rate at which the repeating mechanical waves oscillate between rarefaction and contraction (i.e., the number of cycles per unit of time). Ultrasound encompasses all frequencies above the hearing range for humans (approximately >20kHz), but typically, ranges of frequencies vary according to the biomedical application of ultrasound. Therapeutic applications can operate in lower frequencies, e.g. 20kHz-1MHz, while diagnostic applications usually fall within the 1 MHz - 20 MHz range.

**Mechanical Index (MI)**: a unitless ultrasound metric that can be calculated as:

$$MI= \frac{P_{np}}{\sqrt{f}}$$

Where P_np_ is the Peak Negative Pressure (in MPa) of the wave and f is the driving frequency (in MHz).

**Wavelength (λ)** : The distance traversed by a single cycle of the wave in the medium, which can be calculated by the equation

$$\lambda= \frac{c}{f}$$

where c represents the speed of sound in the medium and f the frequency of the wave. For c = 1500 m/s, which is the approximate speed of sound of water at room temperature, λ can vary from 75 mm to 75 µm for a frequency range of 20 kHz to 20 MHz.

**Reflection angle of incidence:** The angle of incidence of the wave upon the reflecting boundary. The angle of incidence must be < 90゜for a reflection to occur. As the angle of incidence decreases, a smaller portion of the energy is reflected back towards the source.

**Reflection coefficient (R):** For a normally incident wavefront upon a specular reflector, the reflection coefficient R can be calculated by the expression:

$R=\frac{Z_{2}-Z_{1}}{Z_{2}+Z_{1}}$;

where Z_1_ and Z_2_ are the acoustic impedances of media 1 and 2, respectively.

**Supplementary Table S1.** Ultrasound physical phenomena and its corresponding biological effects.

|  | | | | | |
| --- | --- | --- | --- | --- | --- |
| ***Physical phenomenon*** | **Typical in vivo targets and measurement ranges** | **Typical US frequencies** | **Biological effect** | **Application** | **References** |
| ***Reflection*** |  | | | | |
| *Acoustic impedance of object* | Lung, porous tissue: 0.18 x 10^6  MRayls  Bone, dense tissue:7.8 x 10^6 MRayls | 1-20 MHz | Detection of different tissue types, imaging of distinct tissue boundaries and morphology | US imaging of abdominal and pelvic organs, and the brain | [2] |
| *Size of object* | Cells :~15 µm, Rayleigh scatterers  Large organ morphology :~5 cm, specular reflectors | 2-5 MHz (large, deep structures),   5-10 MHz (smaller structures),  10-20 MHz, vasculature, skin, superficial lesions | Detection of different tissue types, imaging of distinct tissue boundaries, vasculature and morphology | US imaging of abdominal and pelvic organs, skin, vasculature, and the brain, speed of sound maps, tissue inhomogeneity | [3] |
| *Velocity of object* | Blood flow in arteries:0.3-1 m/s  Veins: 0.1-0.3 m/s  Microcapillaries: ~1 cm/s | 2-5 MHz (large, deep structures),   5-10 MHz (smaller structures),  10-20 MHz, vasculature, skin, superficial lesions | Measurement of blood flow rate | Diagnosis of blocked arteries, veins, anomalies in vasculature, fetal health monitoring | [4] |
| ***Absorption*** |  | | | | |
| *Attenuation* | Soft tissue: 0.02-1 dB/cm/MHz  Bone: ~20 dB/cm/MHz  Lung: ~40 dB/cm/MHz | 2-5 MHz (large, deep structures),   5-10 MHz (smaller structures),  10-20 MHz, vasculature, skin, superficial lesions | Measurement of elastomechanical properties of tissue | Detection of liver fat, musculoskeletal anomalies, Cellularity of engineered tissue | [5,6] |
| *Shear waves* | Soft tissues, benign tumors: 1-3m/s  Contracted muscles, malignant tumors: >3.5 m/s | 1 kHz (vibrational SWE), ~17 MHz (acoustic radiation force) | Measurement of elastomechanical properties of tissue | Detection of tumor stiffness, skeletal muscle stiffness | [7,8] |
| ***Scattering*** | Size of scatterers ranges from cells to tissue microarchitecture (collagen, fibers) (10-1000 µm) | 1.5-10 MHz | Characterization of tissue microstructure | Detection of osteoporosis, skeletal muscle decomposition, fat build up | [9,10] |
|  | | | | | |
| ***Physical phenomenon*** | **Typical power and duration** | **Typical US frequencies** | **Biological effect** | **Application** | **References** |
| ***Hyperthermia*** |  | | | | |
| *Mild hyperthermia* | 5 - 60 min, 90-150 W/cm2 | tFUS (0.6-0.7 MHz)   soft tissue (1 MHz) | Local heating of tissue upto 40-45 ॰C | Radiosensitization of tumors, targeted drug delivery, neurostimulation | [11,12] |
| *Thermal ablation* | 1-30s, 150-1000 W/cm2 | 0.5-8 MHz, (lower frequencies for deeper tissues) | Rapid heating (>60 ॰C within seconds), thermal necrosis of tissue | Non-invasive surgery for prostate cancer, liver cancer, breast tumors, renal tumors, uterine fibroids | [13] |
| ***Acoustic Radiation Forces*** |  |  |  |  |  |
| *Deformation* | Short pulse (45 µs) | 3-7 MHz | Induces a ‘push’ or deformation in the tissue which varies based on tissue stiffness | ARFI, SWE for staging liver or breast cancer | [14,15] |
| *Translation* | 1-60 minutes | 1-5 MHz | Disruption of tissue structure, transfer of momentum to particle or structure in body | Transdermal transport, propelling drugs across the blood-brain barrier, liquid biopsy | [16,17] |
| *Streaming* | 1-60 minutes | Low MHz | Blood-brain barrier opening, convective effects to promote drug uptake | Transdermal transport | [18] |
| ***Cavitation*** |  | | | | |
| *Assisted cavitation* | 1-60 minutes, 0.5-3 W/cm2 | Any frequencies | Lowers cavitation threshold, enhances permeability of vessel walls, blood brain barrier, improves ultrasound contrast | Targeted delivery of chemotherapeutic agents at site of tumor, transdermal transport of drugs, high-contrast imaging | [19,20] |
| *Stable cavitation* | 1-60 minutes, 0.5-3 W/cm2 | > 1 MHz | Enhance permeability of vessel walls, blood brain barrier, promotes drug release from carriers through localization and convective effects | Targeted delivery of chemotherapeutic agents at site of tumor | [21,22] |
| *Inertial cavitation* | 1-60 minutes, 10-100 W/cm2 | 0.05-1 MHz (sonophoresis),  > 1 MHz (histotripsy) | Disruption of tissue structure | Transdermal drug delivery, lysis of deep-seated clots, blood-brain barrier opening | [23–25] |

**Supplementary References:**

1. Sinden D, ter Haar G. *Transl Cancer Res* https://doi.org/10.3978/j.issn.2218-676X.2014.10.02 (2014) doi:10.3978/j.issn.2218-676X.2014.10.02.

2. Chan, V. & Perlas, A. Basics of Ultrasound Imaging. in *Atlas of Ultrasound-Guided Procedures in Interventional Pain Management* (ed. Narouze, S. N.) 13–19 (Springer New York, New York, NY, 2011). doi:10.1007/978-1-4419-1681-5_2.

3. Jakovljevic, M. *et al.* Local speed of sound estimation in tissue using pulse-echo ultrasound: Model-based approach. *J. Acoust. Soc. Am.* **144**, 254–266 (2018).

4. Nicolaides K, Rizzo G, Hecher K. *Doppler in Obstetrics*.

5. Carroll, D., McKay, L. & Hacking, C. Attenuation (ultrasound). in *Radiopaedia.org* (Radiopaedia.org, 2019). doi:10.53347/rID-67529.

6. Patterson, B. & Miller, D. L. Experimental Measurements of Ultrasound Attenuation in Human Chest Wall and Assessment of the Mechanical Index for Lung Ultrasound. *Ultrasound Med. Biol.* **46**, 1442–1454 (2020).

7. Taljanovic, M. S. *et al.* Shear-Wave Elastography: Basic Physics and Musculoskeletal Applications. *RadioGraphics* **37**, 855–870 (2017).

8. Tsuchida, W. *et al.* Application of the novel estimation method by shear wave elastography using vibrator to human skeletal muscle. *Sci. Rep.* **10**, 22248 (2020).

9. Bi, D. *et al.* The Protocol of Ultrasonic Backscatter Measurements of Musculoskeletal Properties. *Phenomics* **4**, 72–80 (2024).

10. Wang, C.-Y. *et al.* Quantitative imaging of ultrasound backscattered signals with information entropy for bone microstructure characterization. *Sci. Rep.* **12**, 414 (2022).

11. Zhu, L. *et al.* Ultrasound Hyperthermia Technology for Radiosensitization. *Ultrasound Med. Biol.* **45**, 1025–1043 (2019).

12. Chan, H., Chang, H.-Y., Lin, W.-L. & Chen, G.-S. Large-Volume Focused-Ultrasound Mild Hyperthermia for Improving Blood-Brain Tumor Barrier Permeability Application. *Pharmaceutics* **14**, 2012 (2022).

13. Guo, X. *et al.* High-intensity focused ultrasound (HIFU) assisted by a rectal Foley catheter for the treatment of recurrent mucinous ovarian cancer: a case report and literature review. *Front. Oncol.* **14**, 1498631 (2024).

14. Nightingale, K. Acoustic Radiation Force Impulse (ARFI) Imaging: A Review. *Curr. Med. Imaging Rev.* **7**, 328–339 (2011).

15. Doherty, J. R., Trahey, G. E., Nightingale, K. R. & Palmeri, M. L. Acoustic radiation force elasticity imaging in diagnostic ultrasound. *IEEE Trans. Ultrason. Ferroelectr. Freq. Control* **60**, 685–701 (2013).

16. Ciancia, S., Cafarelli, A., Zahoranova, A., Menciassi, A. & Ricotti, L. Pulsatile Drug Delivery System Triggered by Acoustic Radiation Force. *Front. Bioeng. Biotechnol.* **8**, 317 (2020).

17. Li, Y., Guo, M., Guo, G. & Ma, Q. Transdermal drug delivery mediated by acoustic vortex beam. *Ultrasonics* **140**, 107304 (2024).

18. Xu, J. *et al.* Acoustic metamaterials-driven transdermal drug delivery for rapid and on-demand management of acute disease. *Nat. Commun.* **14**, 869 (2023).

19. Vignon, F. *et al.* Microbubble cavitation imaging. *IEEE Trans. Ultrason. Ferroelectr. Freq. Control* **60**, 661–670 (2013).

20. Li, B. *et al.* Low-intensity pulsed ultrasound combined with microbubbles enhances stem cell-based therapy for endometrial injury and intrauterine adhesion. *J. Radiat. Res. Appl. Sci.* **18**, 101319 (2025).

21. McDannold, N., Wen, P. Y., Reardon, D. A., Fletcher, S.-M. & Golby, A. J. Cavitation monitoring, treatment strategy, and acoustic simulations of focused ultrasound blood-brain barrier disruption in patients with glioblastoma. *J. Controlled Release* **372**, 194–208 (2024).

22. Yang, Y. *et al.* Cavitation dose painting for focused ultrasound-induced blood-brain barrier disruption. *Sci. Rep.* **9**, 2840 (2019).

23. Hendley, S. A. *et al.* Clot Degradation Under the Action of Histotripsy Bubble Activity and a Lytic Drug. *IEEE Trans. Ultrason. Ferroelectr. Freq. Control* **68**, 2942–2952 (2021).

24. Goel, L. *et al.* Nanodroplet-mediated catheter-directed sonothrombolysis of retracted blood clots. *Microsyst. Nanoeng.* **7**, 3 (2021).

25. Manikkath, J., Hegde, A. R., Kalthur, G., Parekh, H. S. & Mutalik, S. Influence of peptide dendrimers and sonophoresis on the transdermal delivery of ketoprofen. *Int. J. Pharm.* **521**, 110–119 (2017).
